# Supplementary material for: An endosiRNA-Based Repression Mechanism Counteracts Transposon Activation during Global DNA Demethylation in Embryonic Stem Cells
Source: Cell Stem Cell. 2017 Nov 2;21(5):694–703.e7. doi: 10.1016/j.stem.2017.10.004 (PMC5678422; doi:10.1016/j.stem.2017.10.004)
Supplement: Document S1. Figures S1–S4 and Tables S2 and S3 [file mmc1.pdf]

Cell Stem Cell, Volume *21*

Supplemental Information

An endosiRNA-Based Repression Mechanism Counteracts Transposon Activation during Global DNA Demethylation in Embryonic Stem Cells

Rebecca V. Berrens, Simon Andrews, Dominik Spensberger, Fátima Santos, Wendy Dean, Poppy Gould, Jafar Sharif, Nelly Olova, Tamir Chandra, Haruhiko Koseki, Ferdinand von Meyenn, and Wolf Reik

Supplemental Information

Supplemental Figure Legends

Figure S1. Global DNA demethylation and transcriptional change upon acute Dnmt1 deletion, Related to Figure 1

(A) WGBS-seq reads overlapping the whole Chromosome 2 between WT (day 1-day 11) and *Dnmt1* cKO ESCs induced for 1-11 days. Percentage of methylated cytosines were counted for each consecutive 50 CpG window genome-wide.

(B) Enrichment of CpG methylation over transcription starts sites (TSS) and gene body in WT and *Dnmt1* cKO ESCs induced 1 day (dark red), 3 days (light red), 6 days (light pink), 9 days (light blue), 11 days (dark blue). Measurement of 2 biological replicates. Percentage of methylated cytosines were counted for each consecutive 50 CpG window genome-wide.

(C) Bean plots showing distribution of methylation levels for genome features between WT (gray) and conditional *Dnmt1* cKO ESC induced for 1 day (dark red), 3 days (light red), 6 days (light pink), 9 days (light blue), 11 days (dark blue). Low methylated regions (LMRs) (Stadler et al., 2011), enhancers defined by H3K4m1 (Chen et al., 2012) and H3K27ac (Creyghton et al., 2010). Measurement of 2 biological replicates. For significance analysis Wilcoxon rank sum test with Bonferroni correction testing with a p-value threshold of < 0.05 was used.

(D) Chromosome view of RNA-seq reads over mRNA with Lx5 or MIRb TE sitting in the 2kb surrounding region of a coding gene. RNA-seq libraries are strand specific. Each read is depicted.

(E) Violin plots showing distribution of methylation levels for different TE classes between WT (gray) and conditional *Dnmt1* cKO ESC induced for 1 day (dark red), 3 days (light red), 6 days (light pink), 9 days (light blue), 11 days (dark blue). Measurement of 2 biological replicates. For significance analysis Wilcoxon rank sum test with Bonferroni correction testing with a p-value threshold of < 0.05 was used.

(F) Graphs showing methylation retention of TE classes in comparison to the rest of the genome. Left: scatter plot of WGBS sequencing reads in gradient of gray with specific TE class as red dot, Right: Line graph of TE class in time course (red) in comparison to probes starting with the same methylation level as the respective TE class (blue) and in comparison, to the rest of the genome (gray). Measurement of 2 biological replicates.

(G) Scatter plot of all reads overlapping genes in the genome with the significantly *Dnmt1* responsive genes highlighted in black. Significance was called by combining both Intensity difference (SeqMonk) as well as DESeq2 significance called genes with a p-value threshold of < 0.05 and multiple testing correction.

(H) Venn Diagram of the number and overlap of mRNAs upregulated upon *Dnmt1* cKO.

(I) Bar graph of 6 genes in WT which were most highly upregulated and downregulted upon *Dnmt1* cKO induced 0 days (black), 1 day (dark red), 3 days (light red), 6 days (light pink), 9 days (light blue), 11 days (dark blue). Dots show the expression level in the 2 RNA-seq libraries for each time point.

(J) Bar plots of expression of key pluripotency genes between WT (gray) and conditional *Dnmt1* cKO ESC not induced (black), induced for 1 day (dark red), 3 days (light red), 6 days (light pink), 9 days (light blue). Measurements of 2 biological replicate shown next to each other.

(K) Bar plot showing percentage of genic insertions of *Dnmt1* and *Dicer* responsive TEs in sense (red) and antisense (blue) direction to the respective genes.

Figure S2. Genome wide small RNA response upon Dnmt1 conditional KO, Related to Figure 2

(A) Bar plots of small RNA size distribution as well as classification of different small RNA classes in *Dnmt1* cKO and WT ESCs mapped to the whole genome; miRNAs (gray), rRNA (green), small nuclear RNAs (snRNAs) (violet), miscellaneous other RNAs (misc RNAs) (red), small nucleolar RNAs (snoRNA) (orange) and tRNA (light blue) of WT (right) and after conditional *Dnmt1* cKO (left).

(B) Expression of endogenously transcribed miRNAs in WT (gray) and in conditional *Dnmt1* cKO induced for 1 day (dark red), 3 days (light red), 6 days (light pink), 9 days (light blue), 11 days (dark blue). Error bars represent mean +/-SD of 3 technical replicates.

(C) Genic location of miRNA 200c with reads mapped in Dnmt1 cKO and WT ESCs, each line representing one read.

(D) Scatter plot of all small RNAs in the genome, highlighting miRNAs of the *Dlk* cluster (black) and *Xlr3* cluster (green) at day 9 after *Dnmt1* cKO (y-axis) versus WT (x-axis). Significance was called by combining both Intensity difference (SeqMonk) as well as DESeq2 significance called genes with a p-value threshold of < 0.05 and multiple testing correction.

(E) Bar graph of 2 representative small RNAs of the *Xlr3* and *Dlk* locus in WT and upon *Dnmt1* cKO induced 1 day (dark red), 3 days (light red), 6 days (light pink), 9 days (light blue), 11 days (dark blue). Error bars represent mean +/-SD of 3 technical replicates. Statistics: two-sided Students t-test, * p-value <0.05, ** p-value <0.005, *** p-value <0.0005.

(F) Confirmation of small RNA-seq data by small RNA quantitative real-time PCR analysis. Bar plot showing small RNA quantitative real-time PCR analysis of mmu-miR-543 and mmu-miR-367 in WT (gray) and conditional *Dnmt1* cKO induced for 9 days (dark red). Error bars represent mean +/-SD of 3 technical replicates. Statistics: two-sided Students t-test, * p-value <0.05, ** p-value <0.005, *** p-value <0.0005.

(G) Chromosome view of WGBS-seq, total RNA-seq and small RNA-seq depicted as wiggle plots overlapping imprinted control regions (ICR), mRNA and small RNAs in WT and at day 9 after *Dnmt1* deletion.

(H) Pie chart distribution showing mapping of small RNA-seq from AGO2 IP 9 days after conditional *Dnmt1* to different small RNA classes. miRNAs (black), repeats (dark green), 3'UTRs (yellow), introns (dark blue), piRNAs (light blue), 5'UTRs (light green), others (gray).

(I) Bar plot showing nucleotide position 30 nt upstream and downstream of 5' end of AGO2 IP small RNA-seq libraries mapping to repeats after conditional *Dnmt1* cKO induced 9 days.

(J) Bar plot showing small RNA duplex 5' to 5' overlap of AGO2 IP small RNA-seq mapping to repeats after conditional *Dnmt1* cKO induced 9 days.

(K) Small RNA-seq of 20-24 nt small RNAs mapped to TEs *in vivo* PGCs of E13.5 as well as E14.5 male (blue) and female (red) PGCs. Each library was done as 1 replicate.

(L) Pie chart distribution of small RNAs mapping to different genomic loci of *in vivo* E14.5 male PGC small RNA-seq libraries after conditional *Dnmt1* cKO induced 9 days. miRNAs (black), repeats (dark green), 3'UTRs (yellow), introns (dark blue), 5'UTRs (light green), rRNA_tRNA (gray), unannotated (white).

(M) Size distribution for *in vivo* E14.5 male PGCs of sense (blue) and antisense (red) small RNAs mapping to repeatmasker consensus sequences using piPipes small RNA pipeline.

(N) Bar plot showing siRNA duplex 5' to 5' overlap for *in vivo* E14.5 male PGC small RNA-seq libraries mapping to repeats.

(O) Bar plot showing nucleotide position 30 nt upstream and downstream of 5' end of *in vivo* E14.5 male PGC small RNA-seq library mapping to repeats.

Figure S3. Characterisation of the involvement DICER and AGO2 in TE silencing, Related to Figure 3

(A) Left: Schematic showing *Dicer* cKO generation using CRISPR by introducing loxP sites into Intron 14_15 and Intron 20_21. Agarose gel of PCR to screen for genomic recombination of 2 *Dicer/Dnmt1* conditional double cKO clones after addition of 4OHT for 3 days. Recombination of Intron 15-16 was tested with primer set 1, recombination of intron 20-21 was tested with primer set 2 and recombination of both introns was tested with primer set 3, LD = 1000 bp DNA ladder. Middle: Quantitative real-time PCR analysis of *Dicer* mRNA upon CRE recombination induced by tamoxifen (4OHT) in clone 1 (light green) and clone 2 (dark green) of *Dicer* conditional KO ESCs. Error bars represent mean +/-SD of 3 technical replicates. Statistics: two-sided Students t-test, * p-value <0.05, ** p-value <0.005, *** p-value <0.0005. Right: Quantitative real-time PCR analysis of *mmu-miR-93* expression in ESCs upon *Dicer* KO in clone 1 (light green) and clone 2 (dark green) controlled by snoRNA expression. Error bars represent mean +/-SD of 3 technical replicates. Statistics: two-sided Students t-test, * p-value <0.05, ** p-value <0.005.

(B) Bar graph of percentage of genic antisense transcription over the time course of *Dicer/Dnmt1* cDKO*, Dicer* KO *and Dnmt1* cKO in KO over WT samples. Measurement of 2 biological replicates for *Dicer/Dnmt1* cDKO *and Dnmt1* cKO and WT samples and 1 replicate for *Dicer* KO ESCs.

(C) Bar plots of small RNA size distribution as well as classification of different small RNA classes in *Dicer/Dnmt1* cDKO, *Dnmt1* cKO with KO induced for 4 days and $Dnmt1^{fl/fl}$ mESCs and WT mapped to the whole genome; miRNAs (light blue), rRNA (gray), small nuclear RNAs (snRNAs) (dark blue), miscellaneous other RNAs (misc RNAs) (orange), small nucleolar RNAs (snoRNA) (yellow) and tRNA (light green).

(D) Small RNA-seq of Dicer/Dnmt1 cDKO and Dnmt1 cKO ESCs normalised to WT ESCs mapped to IAPEz and L1MdGf TE classes. *p<0.05, **p<0.005, two-tailed student t-test. Measurement of 2 biological replicates.

(E) Schematic showing *Ago2* cKO generation using CRISPR by introducing loxP sites into Intron 8_9 and Intron 11_12 of *Ago2* mRNA. Agarose gel of PCR to screen for genomic recombination of four *Ago2/Dnmt1* cDKO clones after addition of 4OHT for 3 days in comparison to one WT clone. Recombination of Intron 8-12 was tested with primer set 1. LD = 100 bp DNA ladder.

(F) Quantitative real-time PCR analysis analysis of *Ago2* in ESCs following *Ago2/Dnmt1* cDKO by treatment with 4OHT or control (EtOH) for 3 days. Error bars represent mean +/-SD of 3 biological replicates in 3 technical replicates. Values were normalized to *Hspcb* and controlled to EtOH samples. Statistics: two-sided Students t-test, * p-value <0.05, ** p-value <0.005.

(G) Immunofluorescence of AGO2 protein (purple) in *Ago2/Dnmt1* cDKO and *Dnmt1* cKO ESCs upon KO induction with 4OHT. Deletion was induced for 3 or 8 days as depicted. Nuclear DAPI counter staining (white). scale bar = 20µm.

(H) Upper panel: Schematic knock out strategy for *Dicer* in mouse ESCs constructing gRNAs against Exon 23 and 24 of Dicer mRNA. gRNA Protospacer Adjacent Motif (PAM) sequences (dark blue). (Bernstein et al., 2003). Lower left: Quantitative real-time PCR analysis of mRNA expression of *Dicer* in WT (black) and *Dicer* cKO (dark blue). Error bars represent mean +/- standard deviations of 3 technical replicates. Statistics: two-sided Students t-test, * p-value <0.05, ** p-value <0.005, *** p-value <0.0005. Lower right: Expression level of *mmu-miR-93* in wildtype (black) and *Dicer* cKO (dark blue). Error bars represent mean +/-standard deviations of 3 technical replicates. Statistics: two-sided Students t-test, * p-value <0.05, ** p-value <0.005, *** p-value <0.0005,

(I) Upper panel: Schematic of knock out strategy for *Ago2* in mouse ESCs constructing gRNAs against Intron 13-14 and 115 of Dicer mRNA. gRNA PAM sequences (light green). Lower panel: Quantitative real-time PCR analysis of Ago2 expression in 2 clones of Ago2 KO

ESCs (dark purple) in comparison to Dnmt1$^{fl/fl}$ ESCs (black). Error bars represent mean +/- standard deviations of 3 technical replicates. Statistics: two-sided Students t-test, * p-value <0.05, ** p-value <0.005, *** p-value <0.0005,

(J) Immunofluorescence of AGO2 protein (purple) and NANOG (green) in *Ago2* KO*/Dnmt1* cKO and mouse embryonic fibroblasts. Nuclear DAPI counter staining (white). scale bar = 20µm.

(K) Bar plots of expression of 5 pluripotency genes between WT (gray) and conditional *Dnmt1* cKO ESC induced for 11 days (dark blue), Dicer KO (light blue) treated with EtOH for 1 day and 11 days, Dicer KO/Dnmt1 DKO (faint blue) treated with 4OHT for 1 and 11 days.

(L) Scatter plot of RNA-seq data of *Dicer* KO (y-axis) versus WT (x-axis) ESCs. Differentially expressed genes were called by intensity difference of SeqMonk (black), all other genes are depicted in gray.

(M) Chromosome view of read count quantitation across the 4 genes *Lin28*, *Dnmt3l*, *Fbln2* and *Oct4*. High bars indicated high expression, low bars indicate low expression. Every bar overlaps at least 1 read.

(N) Quantitative real-time PCR analysis data of LINE and major satellites in *Dicer* KO/*Dnmt1* cKO following conditional *Dnmt1* cKO, by treatment with 4OHT. Error bars represent SD of 3 technical replicates. Values were normalized to *Atp5b*, *Hspcb* and Major satellites to U1. Error bars represent mean +/-standard deviations of 3 technical replicates. Statistics: two-sided Students t-test, * p-value <0.05, ** p-value <0.005, *** p-value <0.0005,

(O) Heatmap of unbiased hierarchical clustering of all TE classes responsive *Dicer* KO/*Dnmt1* cKO versus *Dnmt1* cKO. Heatmap is showing relative expression (z-score) of TEs upon *Dnmt1* cKO and were generated using the pheatmap R library.

Figure S4. Distribution of repressive histone marks – H3K9me3, H3K9me2 and H3K27me3 in ESCs upon Dnmt1 cKO, Related to Figure 4

(A) Pie chart of enrichment of H3K27me3, H3K9me3 and H3K9me2 in repeats (dark violet), genic regions (light violet), promoters (dark green), CGIs (middle green), intergenic regions (light green) in wildtype ESCs.

(B) Probe enrichment of H3K9me3 (green), H3K9me2 (yellow) and H3K27me3 (blue) over gene body and TSS in wildtype ESCs.

(C) Aligned probe plot of H3K27me3 enrichment surrounding 5kb of TSS in wildtype ESCs.

(D) Scatter plot of repressive histone marks overlapping genes in wildtype (y-axis) versus *Dnmt1* cKO (x-axis) ESCs.

(E) Read count plots of ChIP enrichment of H3K9me3 (green), H3K27me3 (blue) and H3K9me2 (yellow) over a 500kbp region in Chromosome 12. Intensity of the enrichment on the y-axis. Plots were generated using SeqMonk read count quantitation.

(F) Read count plots of H3K9me3 enrichment over IAPEZ in *Dnmt1* cKO at day 4 (red), day 8 (blue) and in WT (gray). Plots were generated using SeqMonk read count quantitation.
(G) Bar graph of enrichment of H3K27me3, H3K9me3 and H3K9me2 in repeats (dark violet), genic regions (light violet), promoters (dark green), CGIs (middle green), intergenic regions (light green) in WT ESCs, *Dnmt1* cKO, *Dicer* KO and *Dicer/Dnmt1* cDKO
(H) Read count plots of ChIP-seq enrichment of H3K9me3, H3K27me3 and H3K9me2 at three genomic loci in *Dicer/Dnmt1* cDKO at day 11 (light blue), Dicer KO (middle blue), *Dnmt1* cKO at day 11 (dark blue) and WT (gray). Enrichment intensity shown on y-axis. Plots were generated using SeqMonk read count quantitation.
(I) Summary of TE classes across WGBS-seq, RNA-seq, small RNA-seq and ChIP-seq libraries. Scale from red (loose) to green (gain).

Supplemental Tables

Table S1: List of differentially expressed genes upon Dnmt1 KO and Dicer KO, Related to Figure 1 and S1 and Figure 3 and S3.

Differentially expressed genes were called using the overlap between the SeqMonk Intensity difference as well as DESeq2.

Table S2: Quantitative real-time PCR analysis primers, Related to Figure 3 and S3

Primers below have been used for expression analyses (Quantitative real-time PCR analysis primers).

Table S3: CRISPR primers, Related to Figure 3, S3

CRISPR primers were used to construct *Dicer* KO/*Dnmt1* cKO and *Dicer/Dnmt1* cDKO, *Ago2* KO/*Dnmt1* cKO and *Ago2/Dnmt1* cDKO mouse ES cells. gRNA (guide RNA).

Supplemental Data

Data S1: Raw code to analyse TEs, Related to Figure 1-4.

Figure S1

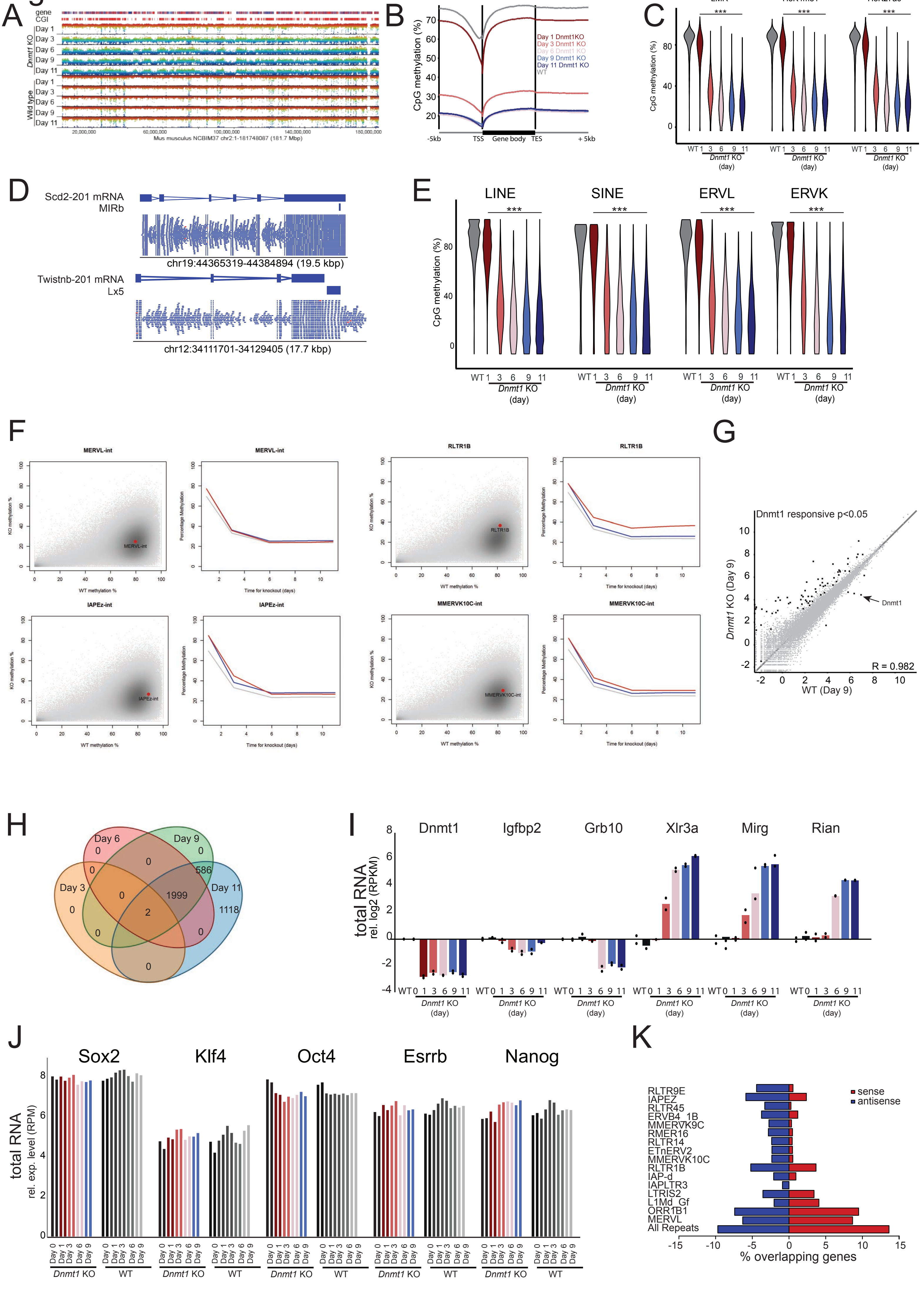
A
gene
CGI
Dnmt1 KO
Wild type
Day 1
Day 3
Day 6
Day 9
Day 11
Mus musculus NCBIM37 chr2:1-181748087 (181.7 Mbp)
B
CpG methylation (%)
Day 1 Dnmt1KO
Day 3 Dnmt1 KO
Day 6 Dnmt1 KO
Day 9 Dnmt1 KO
Day 11 Dnmt1 KO
WT
-5kb
TSS
Gene body
TES
+ 5kb
C
LMR
H3K4me1
H3K27ac
CpG methylation (%)
Dnmt1 KO
(day)
D
Scd2-201 mRNA
MIRb
chr19:44365319-44384894 (19.5 kbp)
Twistnb-201 mRNA
Lx5
chr12:34111701-34129405 (17.7 kbp)
E
LINE
SINE
ERVL
ERVK
CpG methylation (%)
Dnmt1 KO
(day)
F
MERVL-int
RLTR1B
IAPEz-int
MMERVK10C-int
KO methylation %
WT methylation %
Percentage Methylation
Time for knockout (days)
G
Dnmt1 responsive p<0.05
Dnmt1
R = 0.982
Dnmt1 KO (Day 9)
WT (Day 9)
H
Day 3
Day 6
Day 9
Day 11
1999
586
1118
I
Dnmt1
Igfbp2
Grb10
Xlr3a
Mirg
Rian
total RNA
rel. log2 (RPKM)
Dnmt1 KO
(day)
J
Sox2
Klf4
Oct4
Esrrb
Nanog
total RNA
rel. exp. level (RPM)
Dnmt1 KO
WT
K
sense
antisense
RLTR9E
IAPEZ
RLTR45
ERVB4_1B
MMERVK9C
RMER16
RLTR14
ETnERV2
MMERVK10C
RLTR1B
IAP-d
IAPLTR3
LTRIS2
L1Md_Gf
ORR1B1
MERVL
All Repeats
% overlapping genes

Figure S2

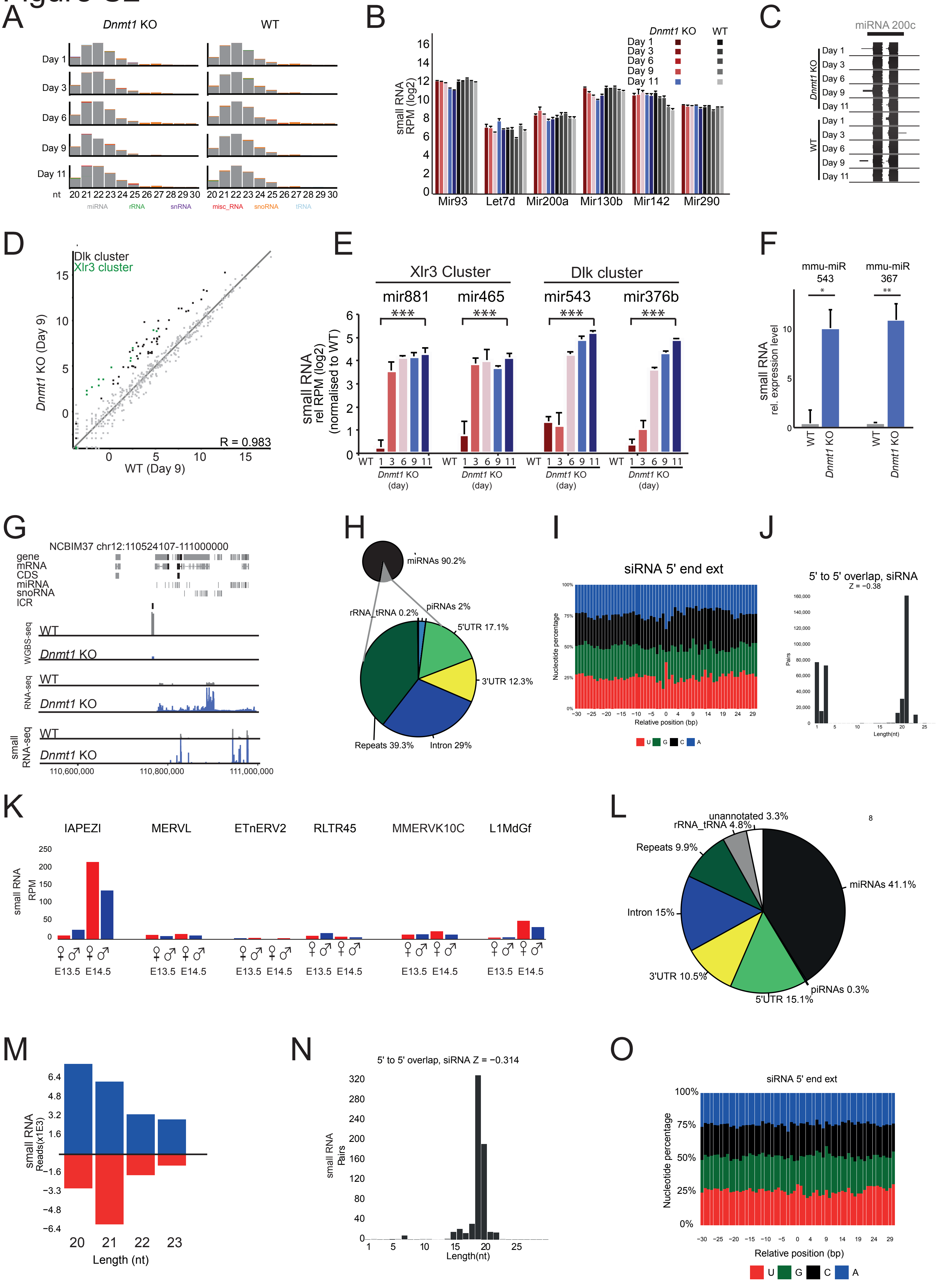
A
Dnmt1 KO
WT
Day 1
Day 3
Day 6
Day 9
Day 11
nt
miRNA rRNA snRNA misc_RNA snoRNA tRNA
B
Dnmt1 KO WT
small RNA RPM (log2)
Mir93 Let7d Mir200a Mir130b Mir142 Mir290
C
miRNA 200c
D
Dlk cluster
Xlr3 cluster
Dnmt1 KO (Day 9)
WT (Day 9)
R = 0.983
E
Xlr3 Cluster
Dlk cluster
mir881 mir465 mir543 mir376b
small RNA rel RPM (log2) (normalised to WT)
Dnmt1 KO (day)
F
mmu-miR 543
mmu-miR 367
small RNA rel. expression level
G
NCBIM37 chr12:110524107-111000000
gene mRNA CDS miRNA snoRNA ICR
WGBS-seq
RNA-seq
small RNA-seq
110,600,000 110,800,000 111,000,000
H
miRNAs 90.2%
rRNA_tRNA 0.2%
piRNAs 2%
5'UTR 17.1%
3'UTR 12.3%
Repeats 39.3%
Intron 29%
I
siRNA 5' end ext
Nucleotide percentage
Relative position (bp)
J
5' to 5' overlap, siRNA
Z = −0.38
Pairs
Length(nt)
K
IAPEZI MERVL ETnERV2 RLTR45 MMERVK10C L1MdGf
small RNA RPM
E13.5 E14.5
L
unannotated 3.3%
rRNA_tRNA 4.8%
Repeats 9.9%
miRNAs 41.1%
Intron 15%
3'UTR 10.5%
piRNAs 0.3%
5'UTR 15.1%
M
small RNA Reads(x1E3)
Length (nt)
N
5' to 5' overlap, siRNA Z = −0.314
small RNA Pairs
Length(nt)
O
siRNA 5' end ext
Nucleotide percentage
Relative position (bp)
U G C A

Figure S3

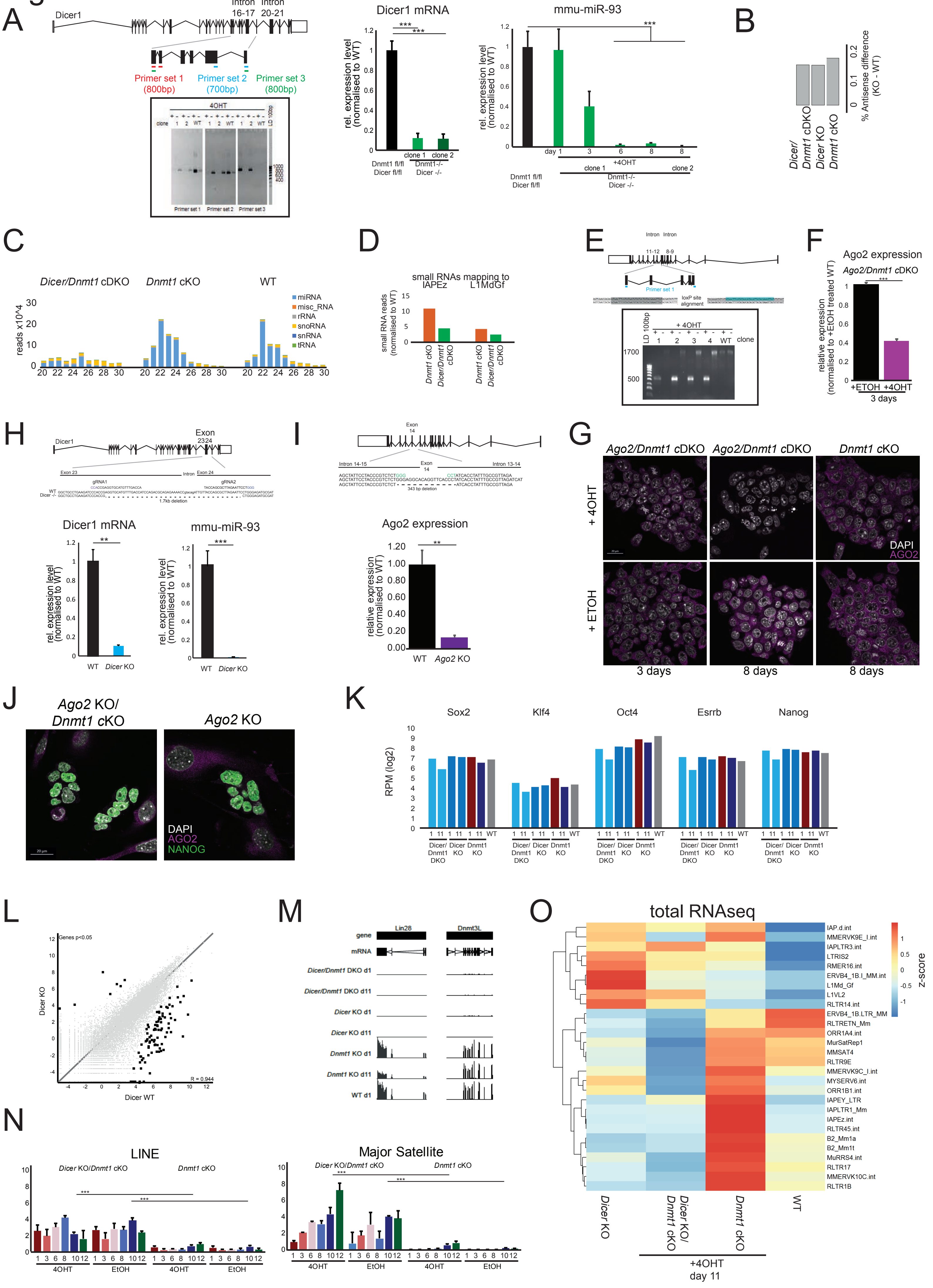
A
Dicer1
Intron 16-17
Intron 20-21
Primer set 1 (800bp)
Primer set 2 (700bp)
Primer set 3 (800bp)
4OHT
clone 1 2 WT 1 2 WT 1 2 WT
LD 100bp
Primer set 1 Primer set 2 Primer set 3
Dicer1 mRNA
rel. expression level (normalised to WT)
Dnmt1 fl/fl Dicer fl/fl
clone 1 clone 2
Dnmt1-/- Dicer -/-
mmu-miR-93
day 1 3 6 8 8
+4OHT
clone 1 clone 2
B
% Antisense difference (KO - WT)
Dicer/ Dnmt1 cDKO
Dicer KO
Dnmt1 cKO
C
Dicer/Dnmt1 cDKO
Dnmt1 cKO
WT
reads x10^4
miRNA
misc_RNA
rRNA
snoRNA
snRNA
tRNA
D
small RNAs mapping to
IAPEz L1MdGf
small RNA reads (normalised to WT)
Dnmt1 cKO
Dicer/Dnmt1 cDKO
E
Intron 11-12
Intron 8-9
Primer set 1
loxP site alignment
+ 4OHT
LD 100bp
clone
F
Ago2 expression
Ago2/Dnmt1 cDKO
relative expression (normalised to +EtOH treated WT)
+ETOH +4OHT
3 days
H
Dicer1
Exon 23 24
Exon 23
Intron
Exon 24
gRNA1
gRNA2
1.7kb deletion
Dicer1 mRNA
mmu-miR-93
rel. expression level (normalised to WT)
WT Dicer KO
I
Exon 14
Intron 14-15
Intron 13-14
343 bp deletion
Ago2 expression
relative expression (normalised to WT)
WT Ago2 KO
G
Ago2/Dnmt1 cDKO
Ago2/Dnmt1 cDKO
Dnmt1 cKO
+ 4OHT
+ ETOH
DAPI
AGO2
3 days
8 days
8 days
J
Ago2 KO/ Dnmt1 cKO
Ago2 KO
DAPI
AGO2
NANOG
20 µm
K
Sox2
Klf4
Oct4
Esrrb
Nanog
RPM (log2)
Dicer/ Dnmt1 DKO
Dicer KO
Dnmt1 KO
WT
L
Genes p<0.05
Dicer KO
Dicer WT
R = 0.944
M
Lin28
Dnmt3L
gene
mRNA
Dicer/Dnmt1 DKO d1
Dicer/Dnmt1 DKO d11
Dicer KO d1
Dicer KO d11
Dnmt1 KO d1
Dnmt1 KO d11
WT d1
N
LINE
Dicer KO/Dnmt1 cKO
Dnmt1 cKO
4OHT EtOH 4OHT EtOH
Major Satellite
O
total RNAseq
IAP.d.int
MMERVK9E_I.int
IAPLTR3.int
LTRIS2
RMER16.int
ERVB4_1B.I_MM.int
L1Md_Gf
L1VL2
RLTR14.int
ERVB4_1B.LTR_MM
RLTRETN_Mm
ORR1A4.int
MurSatRep1
MMSAT4
RLTR9E
MMERVK9C_I.int
MYSERV6.int
ORR1B1.int
IAPEY_LTR
IAPLTR1_Mm
IAPEz.int
RLTR45.int
B2_Mm1a
B2_Mm1t
MuRRS4.int
RLTR17
MMERVK10C.int
RLTR1B
z-score
Dicer KO
Dicer KO/ Dnmt1 cKO
Dnmt1 cKO
WT
+4OHT
day 11

Figure S4

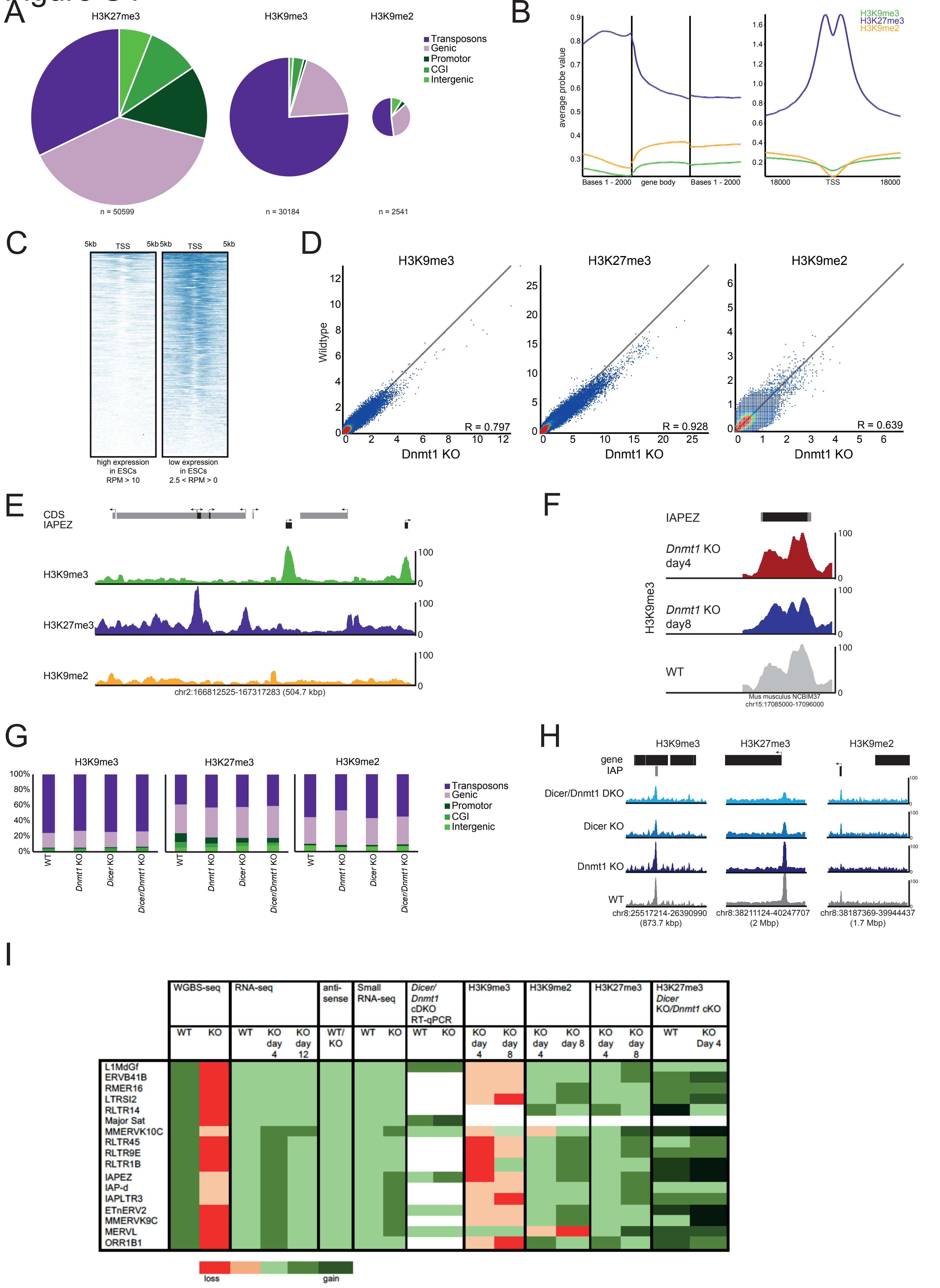
A
H3K27me3
H3K9me3
H3K9me2
Transposons
Genic
Promotor
CGI
Intergenic
n = 50599
n = 30184
n = 2541
B
average probe value
Bases 1 - 2000
gene body
Bases 1 - 2000
18000
TSS
18000
H3K9me3
H3K27me3
H3K9me2
C
5kb
TSS
5kb
high expression in ESCs RPM > 10
low expression in ESCs 2.5 < RPM > 0
D
H3K9me3
H3K27me3
H3K9me2
Wildtype
Dnmt1 KO
R = 0.797
R = 0.928
R = 0.639
E
CDS
IAPEZ
H3K9me3
H3K27me3
H3K9me2
chr2:166812525-167317283 (504.7 kbp)
F
IAPEZ
Dnmt1 KO day4
Dnmt1 KO day8
WT
H3K9me3
Mus musculus NCBIM37
chr15:17085000-17096000
G
H3K9me3
H3K27me3
H3K9me2
WT
Dnmt1 KO
Dicer KO
Dicer/Dnmt1 KO
Transposons
Genic
Promotor
CGI
Intergenic
H
H3K9me3
H3K27me3
H3K9me2
gene
IAP
Dicer/Dnmt1 DKO
Dicer KO
Dnmt1 KO
WT
chr8:25517214-26390990 (873.7 kbp)
chr8:38211124-40247707 (2 Mbp)
chr8:38187369-39944437 (1.7 Mbp)
I
WGBS-seq
RNA-seq
anti-sense
Small RNA-seq
Dicer/Dnmt1 cDKO RT-qPCR
H3K9me3
H3K9me2
H3K27me3
H3K27me3 Dicer KO/Dnmt1 cKO
WT
KO
KO day 4
KO day 12
WT/KO
KO day 8
Day 4
L1MdGf
ERVB41B
RMER16
LTRSI2
RLTR14
Major Sat
MMERVK10C
RLTR45
RLTR9E
RLTR1B
IAPEZ
IAP-d
IAPLTR3
ETnERV2
MMERVK9C
MERVL
ORR1B1
loss
gain

Table S2: RT-qPCR primers, Related to Figure 3 and S3

Gene	Primer	Sequence	origin
Hspcb	msRT_Hspcb_FW	GCTGGCTGAGGACAAGGAGA	
	msRT_Hspcb_RV	CGTCGGTTAGTGGAATCTTCATG	
Atp5b	msRT_Atp5b_FW	GGCCAAGATGTCCTGCTGTT	
	msRT_Atp5b_RV	GCTGGTAGCCTACAGCAGAAGG	
Dicer	Dicer_RT_17_18_FW	GCATTCCTAGCACCAAGTATTCA	This study
	Dicer_RT_17_18_RV	GGAAGGAAATTTACTGAGTGGGG	This study
	Dicer_RT_FW	GAACGAAATGCAAGGAATGGA	
	Dicer_RT_RV	GGGACTTCGATATCCTCTTCTTTCTC	
Ago2	Eif2c2_FW	GCCGTCCTTCCCACTACCAC	
	Eif2c2_RV	GGTATTGACACAGAGCGTGTGC	
Dgcr8	Dgcr8_FW	CCTAAAGACAGTGAAGAACTGGAGTA	
	Dgcr8_RV	CATGGAGGATCTGATATGGAGAC	
IAP	IAP_Nature_qPCR_FW	AAGCAGCAATCACCCACTTTGG	(ref)
	IAP_Nature_qPCR_RV	CAATCATTAGATGTGGCTGCCAAG	(ref)
MERVL	MuERV-L gag_Jafar_FW	TTCTTCTAGACCTGTAACCAGACTCA	(Sharif et al., 2016)
	MuERV-L gag_Jafar_RV	TCCTTAGTAGTGTAGCGAATTTCCTC	(Sharif et al., 2016)
Etn	MusD_Nature_qPCR_FW	GTGGTATCTCAGGAGGAGTGCC	
	MusD_Nature_qPCR_RV	GGGCAGCTCCTCTATCTGAGTG	
U1	U1_AP_FW	CTTACCTGGCAGGGGAGATA	
	U1_AP_FW	CAGTCCCCCACTACCACAAA	
Maj. Sat.	MajSat_BL_FW	GACGACTTGAAAAATGACGAAATC	
	MajSat_BL_RV	CATATTCCAGGTCCTTCAGTGTGC	
MMERVK10C	MmERVK10C_FW	ATGTGAGCTAGCTGTTAAAGAAGGAC	
	MmERVK10C_RV	CTCTCTGTTTCTGACATACTTTCCTGT	
LINEI	LINE ORF2_JS_FW	GACATAGACTAACAAACTGGCTACACAAAC	(Sharif et al., 2016)
	LINE ORF2_JS_RV	GGTAGTGTCTATCTTTTTCTCTGAGATGAG	(Sharif et al., 2016)

Table S3: CRISPR primers, Related to Figure 3 and S3

Gene	Primer	Sequence (5’-3’)	
U6	U6-Fwd	GAGGGCCTATTTCCCATGATTCC	PCR screen
Dicer KO / *Dnmt1* cKO	Dicer1_X23_gRNA_FW	CACCGAGTAATCAAAAGGACCAGCC	gRNA
	Dicer1_X23_gRNA_RV	AAACGGCTGGTCCTTTTGATTACTC	gRNA
	Dicer1_X24_gRNA_FW	CACCGTTACCAGCGCTTAGAATTCC	gRNA
	Dicer1_X24_gRNA_RV	AAACGGAATTCTAAGCGCTGGTAAC	gRNA
	Dicer_23_24_screen_FW	AGCAGTGCATTGCTGACAAGAG	PCR screen
	Dicer_23_24_screen_RV	CTTGTGGTAGTCATACTTCACAGCC	PCR screen
Dicer/Dnmt1 cDKO	Dicer_14_15_gRNA_FW	CACCGCACTCAGCATCGAGTCTCG	gRNA
	Dicer_14_15_gRNA_RV	AAACCGAGACTCGATGCTGAGTGCC	gRNA
	Dicer_20_21_gRNA_FW	CACCGAGCAATGATCCGGTCTCAGG	gRNA
	Dicer_20_21_gRNA_RV	AAACCCTGAGACCGGATCATTGCTC	gRNA
	Dicer_14_15_RV1	TGAAACCAGACTTCTTCAGCTCG	PCR screen
	Dicer_14_15_FW1	CCTTTCCCTCTTGCACATTTACCT	PCR screen
	Dicer_2021_FW1	GGTGTCAGATCACTTCCCGT	PCR screen
	Dicer_2021_RV1	TGACCAGAATAAGAAGGAGCGGA	PCR screen
	Dicer_20_21_donor_loxP	gacaaggaccactgtactgtttatccctgaagtagcagactagaccattgagatcttgtcaagttagagagcagcaagaattctATAACTTCGTATAGCATACATTATACGAAGTTATgagaccggatcattgctcctgtagcagtgatgctggaataggggtgagaatggatatagttcttctcaaaactaa	Donor DNA
	Dicer_14_15_donor_loxP	ggcaagaaaagacatttatttctggttgtggggttaaacaaagcagcagcagcagctcagaaggcactcagcatcgagtctATAACTTCGTATAATGTATGCTATACGAAGTTATcgatcgaagccagagctgcacactgcccaattttacctatgctgcttattacagttttatggaatatcaaaagtatttaaaatag	Donor DNA
Ago2 KO/ *Dnmt1* cKO	Ago2_13_14_gRNA_FW	CACCGCTGGTCTAATCATGATCTAA	gRNA
	Ago2_13_14_gRNA_RV	AAACTTAGATCATGATTAGACCAGC	gRNA
	Ago2_14_15_gRNA_FW	CACCGAAGCTATTCCTACCCGTCTC	gRNA
	Ago2_14_15_gRNA_RV	AAACGAGACGGGTAGGAATAGCTTC	gRNA
	Ago2_13_14_FW	AGGCTACCTTGATGGACATGG	PCR screen
	Ago2_14_15_RV	GATGGGTTTGGTGGTACATGC	PCR screen
Ago2/Dnmt1 cDKO	Ago2_8_9_gRNA_FW	CACCGGTTACCTACAAGTTGTGTG	gRNA
	Ago2_8_9_gRNA_RV	AAACCACACAACTTGTAGGTAACC	gRNA
	Ago2_11_12_gRNA_FW	CACCGGTTGGTCAGACGGGTCACCG	gRNA
	Ago2_11_12_gRNA_RV	AAACAGGGTGACTGCCATTTATGAC	gRNA
	Ago2_8_9_FW	CCTGCTCTTCTGGAGGCATTT	PCR screen
	Ago2_8_9_RV	CCTGCTCTTCTGGAGGCATTT	PCR screen
	Ago2_11_12_FW	GTCCAGGGTGTGTGGGACAT	PCR screen
	Ago2_11_12_RV	GCAACTTCCTCAGCTAATCCTCCA	PCR screen
	Ago2_8_9_donor_loxP	ctcactgtgcacaggtcaagcccagcagagtgccaccaaagctgtagatggtcctctttcatgccagggttacctacaagAtAtCgtATAACTTCGTATAGCATACATTATACGAAGTTATgtgtggttgactttggagtggtcccaccactagtcagggttggttctggcttgcgtactcagcctctgaaatctccct	Donor DNA
	Ago2_11_12_donor_loxP	tgttggtcagacgggtcaccggggttccaataccagcggttggcagcctttctctaacagagagcactcacccaggATAACTTCGTATAGCATACATTATACGAAGTTATgAAtTCgactgccatttatgagatgtgacaaggccagattaggtgtgagagaaaacagctcctgagactgtagaaacttcactgtctat	Donor DNA
