# Supplementary material for: An endosiRNA-Based Repression Mechanism Counteracts Transposon Activation during Global DNA Demethylation in Embryonic Stem Cells
Source: Cell Stem Cell. 2017 Nov 2;21(5):694–703.e7. doi: 10.1016/j.stem.2017.10.004 (PMC5678422; doi:10.1016/j.stem.2017.10.004)
Supplement: Document S2. Article plus Supplemental Information [file mmc4.pdf]

Short Article

Cell Stem Cell

An endosiRNA-Based Repression Mechanism Counteracts Transposon Activation during Global DNA Demethylation in Embryonic Stem Cells

Graphical Abstract

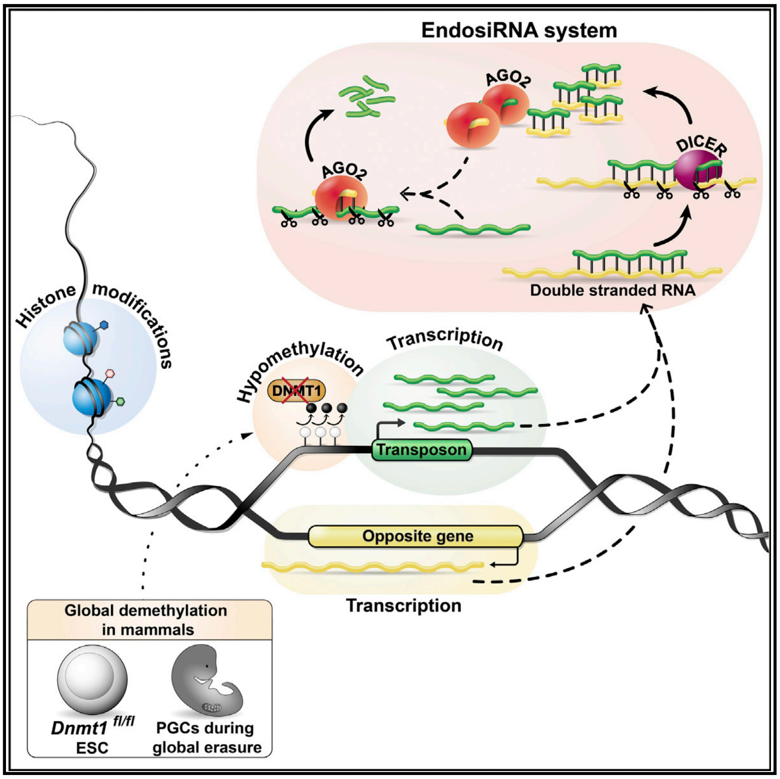

Authors

Rebecca V. Berrens, Simon Andrews, Dominik Spensberger, ..., Haruhiko Koseki, Ferdinand von Meyenn, Wolf Reik

Correspondence

rebecca.berrens@gmail.com (R.V.B.),
vonmeyenn@babraham.ac.uk (F.v.M.),
wolf.reik@babraham.ac.uk (W.R.)

In Brief

In this issue of *Cell Stem Cell*, Berrens et al. report the control of transposable elements by endosiRNAs during global DNA demethylation induced in mouse embryonic stem cells. The study uncovered an "immediate" repression of transposons accomplished by endosiRNAs followed by their "chronic/ long-term" silencing by repressive histone modifications.

Highlights

- Global DNA demethylation in embryonic stem cells leads to transposon activation
- Transposon activation increases the abundance of sense/ antisense transcripts
- ARGONAUTE2-bound endosiRNAs accumulate at high levels for acute repression
- Longer-term transposon repression depends on repressive histone marks

Berrens et al., 2017, Cell Stem Cell *21*, 694–703
November 2, 2017 © 2017 The Authors. Published by Elsevier Inc.
https://doi.org/10.1016/j.stem.2017.10.004

Cell Stem Cell
Short Article

An endosiRNA-Based Repression Mechanism Counteracts Transposon Activation during Global DNA Demethylation in Embryonic Stem Cells

Rebecca V. Berrens,[1,2,6,*] Simon Andrews,[1] Dominik Spensberger,[1] Fátima Santos,[1,2] Wendy Dean,[1] Poppy Gould,[1] Jafar Sharif,[3] Nelly Olova,[1,5] Tamir Chandra,[1,5] Haruhiko Koseki,[3] Ferdinand von Meyenn,[1,*] and Wolf Reik[1,2,4,*]
[1]Epigenetics Programme, Babraham Institute, Cambridge CB22 3AT, UK
[2]University of Cambridge, The Old Schools, Trinity Lane, Cambridge CB2 1TN, UK
[3]RIKEN Research Center for Allergy and Immunology, 1-7-22 Suehiro-cho, Tsurumi, Yokohama, 230-0045 Kanagawa, Japan
[4]Wellcome Trust Sanger Institute, Hinxton CB10 1SA, UK
[5]Present address: MRC Human Genetics Unit, MRC Institute of Genetics and Molecular Medicine, Crewe Road, Edinburgh EH4 2XU, UK
[6]Lead Contact
*Correspondence: rebecca.berrens@gmail.com (R.V.B.), vonmeyenn@babraham.ac.uk (F.v.M.), wolf.reik@babraham.ac.uk (W.R.)
https://doi.org/10.1016/j.stem.2017.10.004

SUMMARY

Erasure of DNA methylation and repressive chromatin marks in the mammalian germline leads to risk of transcriptional activation of transposable elements (TEs). Here, we used mouse embryonic stem cells (ESCs) to identify an endosiRNA-based mechanism involved in suppression of TE transcription. In ESCs with DNA demethylation induced by acute deletion of *Dnmt1*, we saw an increase in sense transcription at TEs, resulting in an abundance of sense/antisense transcripts leading to high levels of ARGONAUTE2 (AGO2)-bound small RNAs. Inhibition of *Dicer* or *Ago2* expression revealed that small RNAs are involved in an immediate response to demethylation-induced transposon activation, while the deposition of repressive histone marks follows as a chronic response. *In vivo*, we also found TE-specific endosiRNAs present during primordial germ cell development. Our results suggest that antisense TE transcription is a "trap" that elicits an endosiRNA response to restrain acute transposon activity during epigenetic reprogramming in the mammalian germline.

INTRODUCTION

Epigenetic reprogramming in the mammalian germline is key for restoration of developmental potency and occurs at the preimplantation stage of embryonic development and during development of primordial germ cells (PGCs) (Reik and Surani, 2015). These events lead to global DNA methylation and H3K9me2 erasure together with the transient transcriptional activation of specific classes of transposable elements (TEs) (Hajkova et al., 2008; Rowe and Trono, 2011). This raises fundamental questions about the regulation of TE defense in the absence of repressive epigenetic marks.

TEs comprise ~50% of the mammalian genome and can be categorized into two major classes: retrotransposons and DNA transposons (Lander et al., 2001). While most TEs in the genome are inactive due to mutations and/or truncations, around 1%–2% of long interspersed nuclear elements (LINEs) and endogenous retroviruses (ERVs) remain able to retrotranspose (Maksakova et al., 2006). Notably, the ERV family members intracisternal A particles (IAPs) and early transposons (ETns) are the most active TEs in the murine germline (Maksakova et al., 2006).

Due to their ability to retrotranspose, TEs are thought to play an important role in genome evolution, but can also cause genetic diseases (Goodier and Kazazian, 2008). In order to protect the genome from harmful mutations, regulatory mechanisms must be in place to limit their transcription.

TE activity is controlled by multiple epigenetic mechanisms including DNA methylation, repressive histone modifications, and small RNAs (Rowe and Trono, 2011). In somatic tissues, DNA methylation and H3K9me2/3 have been shown to be responsible for TE silencing (Walsh et al., 1998; Hutnick et al., 2010). However, in the germline, DNA methylation and H3K9me2 are globally erased, while H3K9me3 is maintained and H3K27me3 is redistributed (Iurlaro et al., 2017; Tang et al., 2016). Indeed, deletion of the H3K9me3 methyltransferase *Setdb1* leads to activation of IAPs during PGC development as well as in mouse embryonic stem cells (ESCs) (Karimi et al., 2011; Maksakova et al., 2006). Further, global demethylation of naive ESCs results in transcriptional activation of TEs and subsequent resilencing by a redistribution of repressive histone marks (Walter et al., 2016).

A number of studies have demonstrated that small RNAs may also act post-transcriptionally as a second-tier defense against TEs, particularly in the germline. In mouse oocytes, microRNAs (miRNAs) and endogenous short interfering RNAs (endosiRNAs) that control TE expression have been identified (Tam et al., 2008; Flemr et al., 2013; Watanabe et al., 2006), and in the male germline PIWI-interacting small RNAs (piRNAs) can also control TE expression (Aravin et al., 2008). In ESCs, tRNA fragments have been recently described to play a role in ERV translational control (Schorn et al., 2017).

In contrast to somatic cells, increased pervasive transcription across TEs was reported in ESCs, suggesting that TEs may regulate transcription of long noncoding RNAs (lncRNAs) (Kelley and Rinn, 2012). Intriguingly, however, in yeast it was shown that

 © 2017 The Authors. Published by Elsevier Inc.
This is an open access article under the CC BY license (http://creativecommons.org/licenses/by/4.0/).

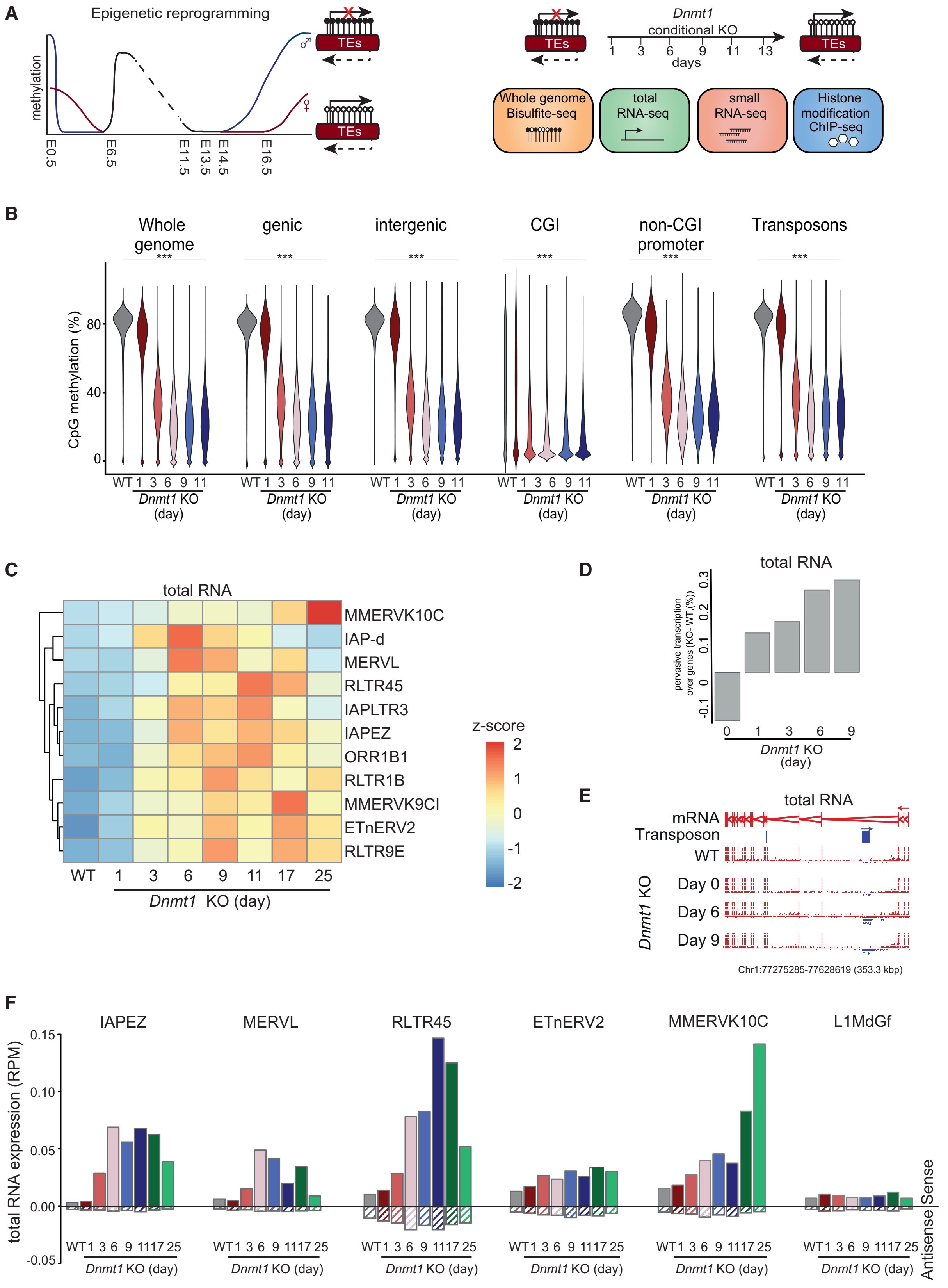

(legend on next page)

genome-wide pervasive transcription antisense to transposons leads to an RNAi response as a defense mechanism against TEs (Cruz and Houseley, 2014). Sense/antisense transcription permits the production of double-stranded RNA (dsRNA) triggering RNAi (Fire et al., 1998), which has also been identified as a control mechanism of TEs (Robert et al., 2005).

Here we test the hypothesis that genic transcripts antisense to TEs serve as a trap for transcriptional activation of TEs during global demethylation in mammals. Generation of *Dicer* as well as *Ago2* conditional and constitutive knockout ESC lines in the background of a *Dnmt1* conditional knockout (cKO) line allowed us to define an "immediate" endosiRNA-dependent repressive response to TE activation and a subsequent "chronic" response, characterized by targeting of repressive histone modifications.

RESULTS

Acute *Dnmt1* Deletion Leads to TE Demethylation in ESCs

Our experimental system recapitulates epigenetic reprogramming of early embryos and PGCs *in vitro*. We used Cre-mediated conditional *Dnmt1* deletion in ESCs (*Dnmt1* cKO) (Sharif et al., 2016) and sampled DNA and RNA at several defined time points after *Dnmt1* deletion for methylome, long and small transcriptome, and chromatin analyses (Figure 1A).

By whole-genome bisulfite sequencing (WGBS-seq), we confirmed that acute deletion of *Dnmt1* led to genome-wide demethylation from an initial 85% CpG methylation to 35% at day 3 after deletion, and 20% at day 6 after deletion with no further demethylation thereafter (Figures 1B and S1A). The residual methylation can be attributed to the activity of the *de novo* DNA methyltransferases (Lei et al., 1996). Upon *Dnmt1* cKO, loss of methylation was observed in genic and intergenic elements, CGIs, and non-CGI promoters (Figure 1B). Characteristic methylation profiles over gene bodies were reduced with the same kinetics as the rest of the genome upon *Dnmt1* cKO (Figure S1B). Furthermore, low methylated regions (LMRs) (Stadler et al., 2011) and active enhancers became demethylated (Figure S1C). Thus, this *in vitro* model results in replication-dependent global demethylation of the genome, which closely resembles the dynamics of global reprogramming in early embryos and PGCs (von Meyenn et al., 2016).

To analyze TEs in WGBS-seq, RNA-seq, and chromatin immunoprecipitation (ChIP)-seq data, we only considered uniquely mapped reads and filtered out TEs overlapping the (± 2 kb) region surrounding genes. While unique mapping might not capture all information about young TEs (as they lack the increased sequence divergence of older TEs that makes unique mapping more efficient; Lerat et al., 2003), this conservative approach allows us to be confident that mapped reads can be definitively ascribed to specific TE subfamilies. Moreover, the filtering of the region (± 2 kb) surrounding genes avoids ambiguity about the origin of TE expression from promoters that are not their own (Figure S1D; Data S1).

Acute *Dnmt1* deletion led to hypomethylation of TEs at the same rate as the rest of the genome (Figures 1B and S1E), with the exception of IAPs, RLTRs, and MMERVK10C, which preserved higher methylation levels (Figure S1F). Thus, our experimental system also closely recapitulates global demethylation dynamics of TEs *in vivo*, including the fact that IAPs are relatively resistant to global demethylation (Seisenberger et al., 2012; Kobayashi et al., 2013).

Increased Sense Transcription of TEs upon Hypomethylation Combines with Pervasive Antisense Transcription

Next, we performed total RNA-seq upon acute *Dnmt1* deletion to examine if demethylation led to transcriptional activation of TEs. Transcriptional activation was limited to specific classes of ERVs (Figure 1C). We found TEs with increased transcription upon hypomethylation that remained active over the whole time course (MMERVK10C), as well as TEs initially activated but notably subsequently re-silenced (e.g., IAPs and MERVLs).

In addition to TEs, a small number of genes became activated upon loss of DNA methylation (Figures S1G and S1H), including the imprinted genes *Xlr3a*, *Mirg*, and *Rian* (Table S1), consistent with the known roles for methylation in regulation of these genes (Ferguson-Smith, 2011) (Figure S1I). DNA hypomethylation did not result in ESC differentiation, as indicated by the unaltered expression of the core pluripotency network (Figure S1J).

Interestingly, when quantifying reads overlapping with genes, we found upon global hypomethylation increased pervasive transcription in the antisense orientation to those genes (Figure 1D). These pervasive antisense transcripts are in fact produced by transcription of TEs that have integrated in an antisense orientation to the genes (Figure 1E). Consistent with previous studies, high numbers of TEs were found to be preferentially integrated in antisense orientation to genes (van de Lagemaat et al., 2006) (Figure S1K).

Figure 1. Transcriptional Upregulation of Specific TE Classes upon Acute *Dnmt1* Deletion

(A) Left: schematic overview of epigenetic reprogramming during preimplantation and male (blue) and female (red) germline development. Right: schematic of *Dnmt1* cKO as an *in vitro* system for mechanistic study of TE regulation during epigenetic reprogramming.

(B) Violin plots showing the distribution of CpG methylation levels measured by WGBS-seq of WT (gray) and conditional *Dnmt1* cKO ESCs induced for days depicted in the figure. The percentage of methylated cytosines was quantified in consecutive 50 CpG windows genome-wide. CGI, CpG island. For significance analysis, Wilcoxon rank-sum test with Bonferroni correction testing with a p value threshold of <0.05.

(C) Heatmap of unbiased hierarchical clustering of all TEs responsive to *Dnmt1* cKO across the time course of KO induction. The relative expression (*Z* score) of TEs upon *Dnmt1* cKO is shown; n = 2.

(D) Bar graph showing the percentage of genic antisense transcription upon *Dnmt1* deletion in KO relative to WT samples; n = 2.

(E) Chromosome view of TE inserted antisense to gene. Position of TE is denoted (top panel) along with sense strand-specific RNA-seq reads (lower panels; sense transcription shown in blue; antisense transcription shown in red). Each read is depicted. Arrows indicate directionality of reads.

(F) Expression of TEs in conditional *Dnmt1* cKO ESC. Shown are normalized RNA-seq read counts overlapping different TE classes in sense (filled bars) or antisense (hatched bars) orientation. The figure shows mean of n = 2.

See also Figures S1 and S4I and Data S1.

We next analyzed the total RNA-seq data to determine whether both sense and antisense transcription was detectable at sites of TE integration. Indeed, TE antisense transcription was found in all TE families, with sense transcripts of members of the ERVs being upregulated consistent with their activation in response to demethylation (Figure 1F). We also included TEs that were not activated by hypomethylation, but instead are regulated in a DICER-dependent manner (Figure 3E).

Sense/Antisense Transcription of TEs Correlates with AGO2-Bound endosiRNAs

The production of sense and antisense transcripts across TEs is expected to lead to dsRNAs, which can subsequently induce an RNAi response and silence TEs post-transcriptionally. These results suggest that TE expression may be sensed by pervasive antisense transcription, thus constituting a TE "trap" (Figure 2A). To test this hypothesis, we performed small RNA-seq at defined time points after *Dnmt1* deletion. The majority of small RNAs were miRNAs (Figures S2A–S2C) and were expressed independently of DNA methylation, with the exception of miRNAs from the imprinted *Dlk* and *Xlr3* loci (Figures S2D and S2E). Small RNA quantitative real-time PCR of mature miRNAs confirmed their methylation-dependent regulation (Figure S2F). The *Dlk* locus serves as an example of the genome-wide response to acute *Dnmt1* deletion with the imprint control region (ICR) becoming demethylated, leading to transcriptional upregulation of the imprinted locus and embedded miRNAs (Figure S2G).

Due to the short reads in small RNA-seq, we used TE consensus sequence mapping to analyze global TE-derived small RNAs. This method allows unambiguous alignment to unique TE classes. Notably, we observed a substantial increase of small RNAs mapping to IAP, MERVL, and ETn upon *Dnmt1* deletion (Figure 2B), which in the case of IAPs mapped across the whole length of the element (Figure 2C). Small RNAs mapping to L1MdGf and MMERVK10C were detected both in wild-type (WT) and *Dnmt1* cKO ESCs, respectively (Figure 2B).

The mammalian ARGONAUTE proteins (AGO) are critical components of the RNA-induced silencing complex (RISC). AGO2 can bind miRNAs as well as endosiRNAs and has the ability to "slice" its targets (Doi et al., 2003). We performed AGO2 IP from *Dnmt1* cKO ESCs at day 9 after deletion and analyzed the pull-down by small RNA-seq (Figure 2D). The AGO2 IP small RNA-seq libraries of both WT and *Dnmt1* cKO ESCs were composed 90% of known miRNAs, while 40% of the remaining small RNAs mapped to TEs (Figure S2H, *Dnmt1* cKO shown). This subset of AGO2-bound small RNAs was 22 nt long and mapped to sense and antisense strands of TEs (Figure 2E); the small RNAs had 5′ U overhangs (Figure S2I) and formed characteristic 5′-5′ overlaps at nucleotide 20, identifying them as bona fide endosiRNAs (Figure S2J) (Ghildiyal and Zamore, 2009). AGO2-bound endosiRNAs mapping to MERVL and RLTR45 were expressed throughout the time course while endosiRNAs mapping to L1, IAP, and ETn or MMERVK10C were significantly enriched upon *Dnmt1* deletion (Figure 2F), suggesting that functional endosiRNAs against specific TE classes are generated during global demethylation.

We also generated small RNA-seq libraries of male and female PGCs from embryonic day (E)13.5 and E14.5 embryos and found that ~10% of all 20–24 nt small RNAs mapped to TEs in both male and female E13.5 and E14.5 PGCs, with small RNAs mapping to IAPEZ and L1MdGf particularly enriched in E14.5 PGCs (Figures S2K and S2L). These small RNAs had the defining properties of endosiRNAs (Figures S2M–S2O), suggesting that a similar response to the one we discovered in ESCs exists during global demethylation in the germline *in vivo*.

Key RNAi Components Are Involved in the Repression of Specific TE Classes

To investigate whether the observed endosiRNAs were involved in restraining TE expression, we knocked down key components of the endosiRNA and miRNA pathways in *Dnmt1* cKO and monitored IAP expression by quantitative real-time PCR. Upon knockdown of *Dicer* or *Ago2*, both essential components of the RNAi pathway, IAP transcription was strongly upregulated, while knockdown of *Dgcr8* (dispensable for endosiRNA function) had no effect on IAP expression (Figure 3A). This suggests that TEs are controlled by functional endosiRNAs.

To examine the role of the RNAi pathway during global hypomethylation in more detail, we generated conditional *Dicer*/*Dnmt1* cDKO (conditional double-knockout) ESCs (Figure S3A) and carried out a number of quality controls. Loss of *Dicer* activity was confirmed by loss of expression of mmu-miR-93, while *Dicer*-independent small nucleolar RNAs (snoRNAs) were still expressed (Figure S3A). We generated total RNA-seq data from *Dicer*/*Dnmt1* cDKO ESCs and found increased antisense transcripts in these cells, as seen earlier in the *Dnmt1* cKO ESCs (Figure S3B). Furthermore, small RNA-seq of *Dicer*/*Dnmt1* cDKO ESCs showed a depletion of all miRNAs (Figure S3C) and a loss of 21–24 nt small RNAs mapping to all TEs as well as specifically to L1MdGf and IAPEz (Figures 3B and S3D), which proves that the described small RNAs are DICER-dependent products.

Acute conditional deletion of both *Dicer* and *Dnmt1* together resulted in significantly higher levels of transcription of IAPs by day 10 in comparison to those in *Dnmt1* cKO ESCs (Figure 3C). Importantly, there was no notable resilencing of IAP transcripts in *Dicer*/*Dnmt1* cDKO. This demonstrates that DICER plays a role in the re-repression of IAPs upon global hypomethylation. LINEs and major satellites (non-TE pericentric repeats), while not upregulated upon *Dnmt1* deletion, were also dramatically upregulated following *Dicer* deletion (Figure 3C). *Dicer*/*Dnmt1* cDKO ESCs started to show signs of cell death from day 12 after deletion, potentially as a result of TE mobilization, as has been shown in constitutive *Dicer* KO (Bodak et al., 2017).

We next asked whether deletion of RNAi components downstream of DICER would lead to a similar response and generated conditional *Ago2*/*Dnmt1* cDKO ESCs (Figure S3E). While we initially expected that *Ago2*/*Dnmt1* cDKO might show comparable results to the *Dicer*/*Dnmt1* cDKO, we found that the deletion kinetics of *Ago2* KO were substantially slower than those of *Dicer* KO (Figures S3F and S3G). Surprisingly, however, we found that transcriptional upregulation of TEs in the *Ago2*/*Dnmt1* cDKO was considerably blunted (Figure 3D).

To gain deeper insights into the blunted TE expression, we constitutively deleted *Ago2* or *Dicer* using CRISPR/Cas9 genome editing in the background of *Dnmt1* cKO ESCs (Figures S3H–S3J). We first determined the effect of *Dicer* KO on genic and transposon transcription and were able to identify

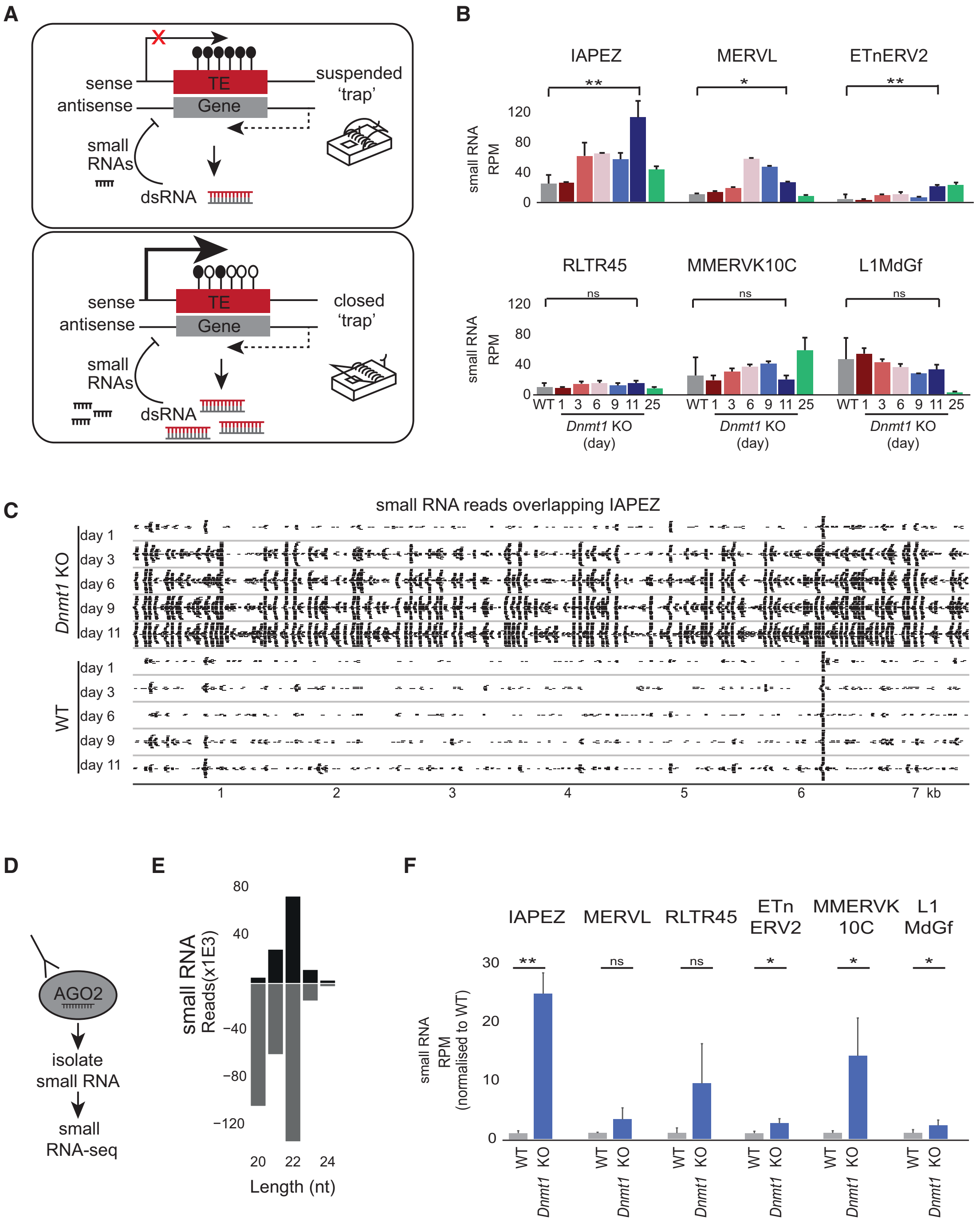

Figure 2. Generation of TE-Derived Small RNAs following Global Demethylation

(A) Schematic displaying the hypothesis of pervasive transcription overlapping TEs acting as a "trap" of transcriptional activation of TEs. This could work through the production of dsRNAs from sense and antisense transcripts that feed into the RNAi pathway, which subsequently silences the TEs.

(B) Small RNA-seq reads mapped to different classes of TEs from WT (gray) and conditional *Dnmt1* cKO ESCs. *$p < 0.05$, **$p < 0.005$, two-tailed Student's t test. Bars represent mean ± SD, n = 3. All reads of a size between 20 and 24 nt were mapped to TE consensus sequences.

(C) Small RNA-seq reads mapped to the consensus sequence of IAPEZ. All reads of a size between 20 and 36 nt were mapped to the IAPEZ consensus sequence.

(D) Schematic displaying AGO2 IP of small RNAs.

(E) Size distribution of AGO2-bound small RNAs after AGO2 IP of sense (black) and antisense (gray) small RNAs mapping to repeatmasker consensus sequences using the piPipes small RNA-seq pipeline (Han et al., 2015).

(F) Small RNA-seq of AGO2-bound small RNAs mapped to TE classes of WT (gray) and conditional *Dnmt1* cKO ESCs induced for 9 days (light blue). *$p < 0.05$, **$p < 0.005$, two-tailed Student's t test. Bars represent mean ± SD, n = 4.

See also Figures S2 and S4I and Data S1.

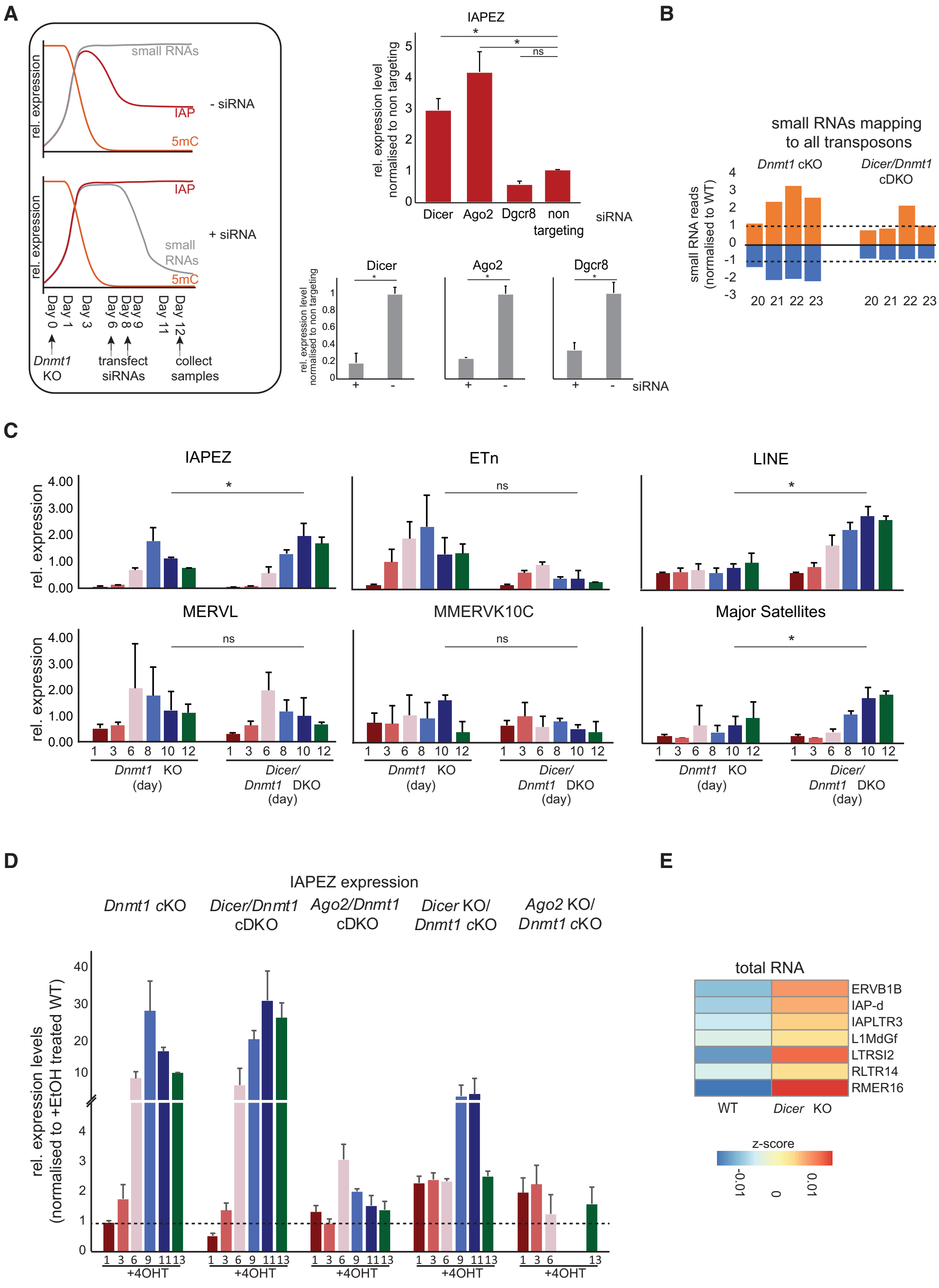

Figure 3. TEs Are Repressed by a DICER Mechanism

(A) Knockdown (KD) of RNAi players. Left: schematic of siRNA KD in *Dnmt1* cKO ESCs. The genome gets demethylated (5mC, orange) and IAPs get transcriptionally activated and resilenced (red) if small RNAs are present (gray); however, KD of the RNAi pathway will deplete small RNAs. Lower right: quantitative real-time PCR analysis showing KD efficiencies of *Dicer*, *Ago2*, and *Dgcr8* upon treatment with siRNAs after *Dnmt1* deletion. Upper right: expression of IAPs upon *Dicer*, *Ago2*, *Dgcr8*, or non-targeting siRNA transfection. The data are normalized to non-targeting control. Bars represent mean ± SD, n = 3. $^{*}p < 0.05$, $^{**}p < 0.005$, two-tailed Student's t test.

(legend continued on next page)

TEs that were solely dependent on DICER for their silencing, such as L1MdGf (Figures 3E and S3K–S3O).

We next performed a time course of *Dnmt1* deletion in *Ago2* KO/*Dnmt1* cKO and in *Dicer* KO/*Dnmt1* cKO and measured IAP expression by quantitative real-time PCR. Notably, we found substantially attenuated upregulation of IAPs upon *Dnmt1* deletion in both ESC lines, which was confirmed by total RNA-seq (Figures 3D and S3O). These results indicate that, in addition to DNA methylation and RNAi, alternative TE silencing mechanisms can be recruited. While DICER-dependent mechanisms restrict the expression of specific TE classes upon deletion of *Dnmt1*, ablation of the RNAi pathway prior to demethylation triggers the engagement of another silencing mechanism. Since repressive histone marks have been shown to contribute to TE repression in somatic tissues and in ESCs (Karimi et al., 2011; Maksakova et al., 2006; Walter et al., 2016), we asked whether these might constitute the additional repressive mechanism observed here.

TE Silencing by Repressive Histone Marks

To study the involvement of chromatin in TE regulation upon global hypomethylation, we carried out ChIP-seq analyses of the repressive histone marks H3K9me2, H3K9me3, and H3K27me3 at 4 and 8 days after deletion of *Dnmt1*, i.e., before and after transcriptional upregulation of the relevant TE classes. Genome-wide distribution of the repressive histone marks—H3K27me3, H3K9me2, and H3K9me3—confirmed earlier studies (Iurlaro et al., 2017; Tang et al., 2016) with H3K27me3 enrichment in gene bodies and H3K9me2/3 enrichment in TEs (Figure S4A). Additionally, H3K27me3 was enriched in promoter regions but depleted at transcription start sites (TSSs) (Figures S4B and S4C). Upon *Dnmt1* deletion, neither of these repressive histone marks were redistributed genome-wide (Figure S4D).

However, DICER-independent MERVLs showed increased H3K27me3 deposition upon *Dnmt1* deletion, recapitulating what has been reported in naive hypomethylated ESCs (Walter et al., 2016) (Figure 4A). We found H3K9me3 enrichment across IAPs independent of DNA methylation levels, confirming previous results (Figures S4E and S4F) (Walter et al., 2016; Sharif et al., 2016). Importantly, H3K27me3 and H3K9me2 deposition was found on IAPs 9 days after *Dnmt1* deletion, explaining why early, but not late, depletion of *Dicer* or *Ago2* results in sustained TE expression. These results show that two repressive pathways are in place to control TE expression in ESCs (Figure S4I), and importantly, that they are staggered in time, with an "immediate" RNAi response and a subsequent "chronic" chromatin response.

To obtain insights into the attenuated IAP expression in *Dicer* KO/*Dnmt1* cKO, we performed ChIP-seq of the same repressive histone marks. While we did not observe a genome-wide redistribution of H3K27me3, H3K9me2, and H3K9me3 in the *Dicer* KO or the *Dicer* KO/*Dnmt1* cKO (Figures S4G and S4H), we observed a clear redistribution of repressive histone marks over TEs in *Dicer* KO and in particular an enrichment of H3K27me3 and of H3K9me2 at IAPs. This was even further increased upon *Dnmt1* deletion (Figure 4B). Hence, acute deletion of *Dicer* during global demethylation abrogates re-silencing of IAPs while constitutive deletion of *Dicer* instigates a repressive chromatin response in IAPs that suppresses reactivation upon hypomethylation (Figure 4C).

DISCUSSION

How TEs are controlled during global epigenetic reprogramming in the mammalian germline is a highly relevant question. The present study provides, to our knowledge, the first evidence of AGO2-bound endosiRNAs in ESCs during global DNA hypomethylation, which restrict TE expression as judged by acute depletion of *Dicer* or *Ago2*. As we also detect DICER-dependent endosiRNAs in PGCs, it is likely that the described mechanism also operates *in vivo*. This mechanism constitutes a first line of TE defense during epigenetic reprogramming. A second line of defense is provided by chromatin targeting and retargeting, presumably through the evolution of sequence-specific recognition modules of TEs such as zinc-finger proteins (Rowe and Trono, 2011). Our work also indicates a link between these systems; they are staggered in time and thus potentially connected.

Many TE families are associated with transcribed genes or lncRNAs in ESCs (Kelley and Rinn, 2012). This provides the potential for sense/antisense transcription to occur when TEs become demethylated, as observed here (Figure 1F). In oocytes, pseudogenes provide the antisense strand to TEs to feed into an RNAi pathway (Tam et al., 2008) and TEs have been shown to give rise to dsRNA in preimplantation embryos due to their bidirectional promoters (Svoboda et al., 2004). Indeed, we found intragenic active TEs preferentially integrated in antisense direction to the gene (Figure S1K). Previous studies had concluded that this could prevent disruption of normal gene expression (van de Lagemaat et al., 2006). We suggest an additional reason why this direction of insertion is evolutionarily favored: it produces a trapping system ("trap") for transposon activation during epigenetic reprogramming, in order to tame newly invading TEs (Figure 2A).

Overlapping sense/antisense transcription feeds into an endosiRNA pathway regulated by DICER and AGO2 to silence TEs.

(B) Small RNA-seq of *Dicer*/*Dnmt1* cDKO and *Dnmt1* cKO ESCs. Sense (orange) and antisense (blue) small RNAs are separated by size and were mapped to all TEs. Reads were normalized to non-induced WT (*Dicer* $^{fl/fl}$ /*Dnmt1* $^{fl/fl}$) ESCs.

(C) Quantitative real-time PCR analysis of TE classes in ESCs following conditional *Dnmt1* cKO or *Dnmt1*/*Dicer* cDKO by treatment with 4OHT or *Dicer* KO. Bars represent mean of two biological replicates with two technical replicates. Values were normalized to *Atp5b* and *Hspcb*, and major satellites were normalized to U1. *$p < 0.05$, **$p < 0.005$, two-tailed Student's t test.

(D) Quantitative real-time PCR analysis of IAPEz in the indicated ESC lines. Conditional deletions were induced by treatment with 4OHT for the indicated days. Values were normalized to *Atp5b* and *Hspcb* and are relative to the respective WT sample for each KO line, indicated by dashed line. Error bars represent mean ± SD; n = 3 for *Dnmt1* cKO, *Dicer* KO/*Dnmt1* cKO, and *Ago2* KO/*Dnmt1* cKO; n = 2 for *Dicer*/*Dnmt1* cDKO and *Ago2*/*Dnmt1* cDKO. *Ago2* KO/*Dnmt1* cKO time points days 9 and 11 were not collected.

(E) Heatmap of unbiased hierarchical clustering of all TE classes responsive to *Dicer* KO. Heatmap depicts relative expression (*Z* score) of TEs upon *Dicer* KO. See also Figures S3 and S4I and Tables S2 and S3.

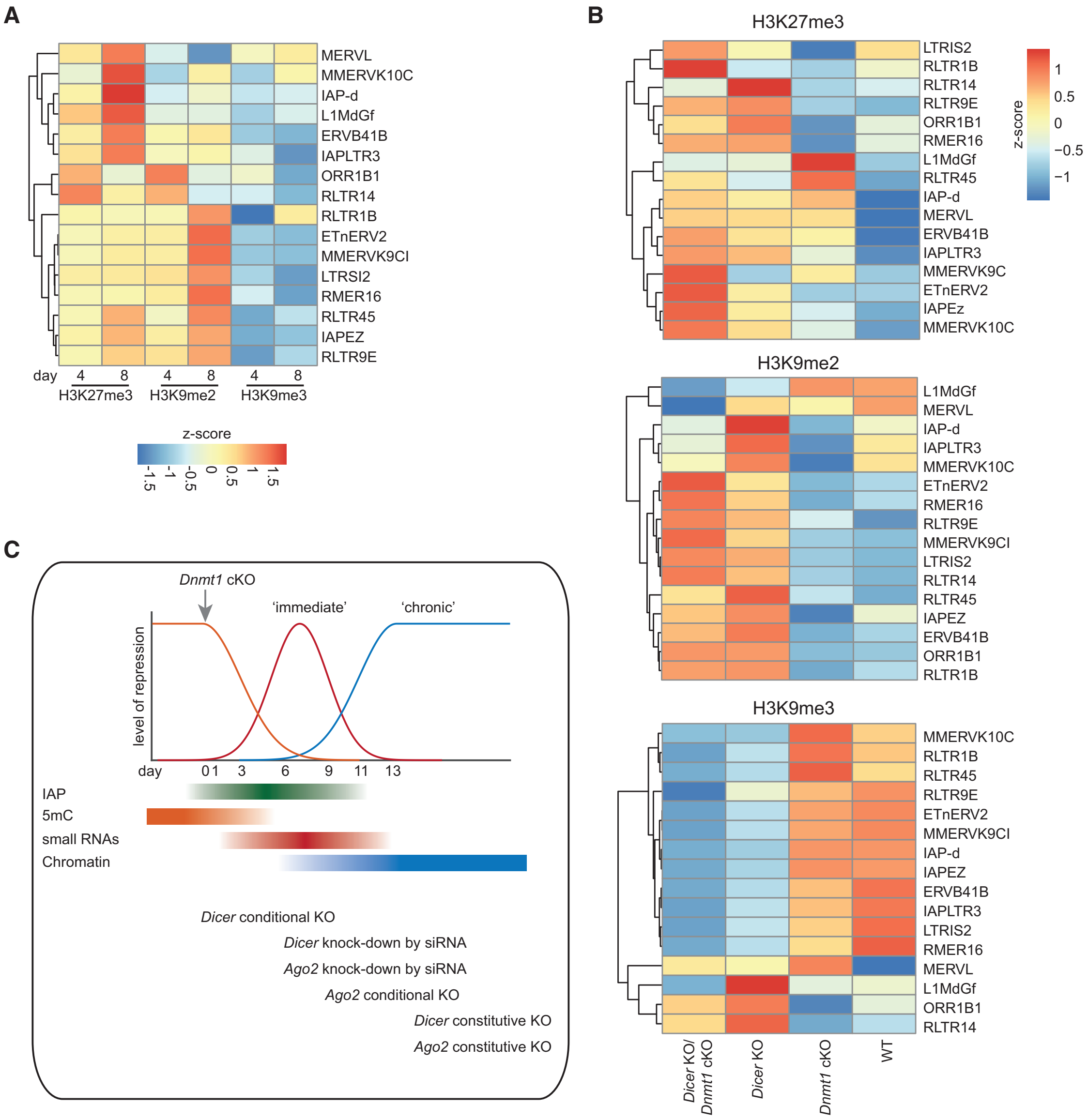

Figure 4. Repressive Histone Modifications Enriched at TEs upon Global Demethylation

(A) Heatmap showing relative enrichment (*Z* score) of repressive histone marks (H3K9me3, H3K27me3, and H3K9me2) at TE classes differentially regulated upon both *Dicer* KO (Figure 3A) and *Dnmt1* cKO (Figure 1C) and normalized to enrichment in WT ESCs upon acute deletion of *Dnmt1*.

(B) H3K27me3, H3K9me3, and H3K9me2 enrichment over TEs dependent on *Dicer* and *Dnmt1*. Heatmap depicts ChIP-seq data of H3K27me3 mapped to TE families at depicted days after *Dnmt1* cKO, *Dicer* KO, and *Dnmt1*/*Dicer* cDKO in comparison to WT ESCs.

(C) Schematic of the two levels of TE control upon global demethylation. Upon deletion of *Dnmt1*, DNA methylation (5mC; orange)-mediated repression is lost, and transposon expression increases (as an example, IAP expression is shown in green). Subsequently, small RNAs (red; "immediate" response) and repressive histone marks (chromatin, blue; "chronic" response) establish a new repressive environment. Also indicated are the time points at which the different experimental manipulations interfere with the system.

See also Figure S4 and Data S1.

The generation of the two constitutive and conditional KO ESCs in the background of the *Dnmt1* cKO allowed us to dissect the dynamics of TE control during global hypomethylation, revealing an "immediate" response that is characterized by endosiRNAs and affected by acute depletion of *Dicer* or *Ago2*. This is followed by a "chronic" response, which is defined by targeting of repressive histone modifications (particularly H3K27me3 and H3K9me2) and occurs subsequent to the endosiRNA response in *Dnmt1* cKO and *Dnmt1*/*Dicer* cDKO ESCs (Figure 4C). Intriguingly, non-acute depletion of *Dicer* also instigates deposition of H3K27me3 and H3K9me2 independently of DNA demethylation, suggesting that the two systems are linked. We suggest a mechanism of TE control by which the "immediate" endosiRNA response to global methylation erasure is followed by a "chronic" repressive chromatin response. Interestingly, the "chronic" response is initiated by deletion of *Dnmt1* as well as by abrogation

of the "immediate" defense. Therefore, the "immediate" and "chronic" responses are not only staggered in time, but also appear mechanistically linked. Unravelling the molecular underpinnings of this link will be an important topic of future work.

The specific response of IAPs and LINEs to loss of DICER may be explained by the fact that they embody the most active retrotransposition competent TE copies in the mouse germline (Maksakova et al., 2006) and are primarily guarded by endo-siRNAs, with chromatin playing a secondary role in their transcriptional restriction. Other TEs, in contrast, are primarily controlled by chromatin redistribution upon global demethylation. The present study highlights the exquisite variety and interplay of epigenetic modifications by which the transcription of different TE families is regulated. Future work in this area, particularly with high-coverage long-read sequencing, will hopefully allow the characterization of transcriptional and epigenetic regulation of individual TE copies in the genome.

We identified DICER as an important factor in small RNA-dependent silencing of TEs. Nonetheless, DICER-independent AGO2-bound small RNAs may also play a role in TE silencing (Babiarz et al., 2008; Murchison et al., 2005). DICER-independent small RNAs might also explain the repression of ETns, to which increasing amounts of AGO2-bound small RNAs mapped, but which were not responsive to *Dicer* KO.

TEs benefit from transcriptional activation in the germline, but not in somatic cells (Haig, 2016). Hence, one might speculate that they may regulate aspects of epigenetic reprogramming in germ cells to their benefit. In this respect, TEs may not be the sole benefactors of their own mobilization, but it also impacts the creation of novelty in the host genome. Nevertheless, unrestrained activation and transposition would presumably be detrimental to the host genome, and hence a sophisticated balance of regulatory mechanisms for TEs has evolved in the germline, including the chromatin retargeting and the endosiRNA pathway we report here.

STAR★METHODS

Detailed methods are provided in the online version of this paper and include the following:

- KEY RESOURCES TABLE
- CONTACT FOR REAGENT AND RESOURCE SHARING
- EXPERIMENTAL MODEL AND SUBJECT DETAILS
  - Cell lines
  - Mice
- METHOD DETAILS
  - DNA/RNA Extraction
  - Small RNA Quantitative Real-Time PCR
  - AGO2 IP
  - RNAi knockdown of Ago2, Dicer1, Dgcr8 in Dnmt1fl/fl ES cells
  - Quantitative Real-Time PCR
  - CRISPR cKO and KO
  - Fluorescence-activated cell sorting (FACS)
  - CD4 pull down
  - *In vivo* PGC collection
  - Cell lines and culture conditions
  - WGBS-seq libraries
  - ChIP-seq libraries
  - Small RNA-seq libraries
  - Total RNA-seq libraries
- QUANTIFICATION AND STATISTICAL ANALYSIS
  - WGBS-seq mapping and analysis
  - RNA-seq mapping and analysis
  - ChIP-seq mapping and analysis
  - Small RNA-seq mapping and analysis
  - Transposon analysis
  - Statistics
- DATA AND SOFTWARE AVAILABILITY

SUPPLEMENTAL INFORMATION

Supplemental Information includes four figures, three tables, and one data file and can be found with this article online at https://doi.org/10.1016/j.stem.2017.10.004.

AUTHOR CONTRIBUTIONS

R.V.B. conceived and designed the study, performed experiments, analyzed data, and wrote the paper; S.A. analyzed data; D.S., W.D., P.G., J.S., and F.S. performed experiments; N.O. and T.C. helped to design the project; J.S. and H.K. generated original conditional *Dnmt1* knockout ESCs; F.v.M. designed and supervised the study and wrote the paper; and W.R. conceived, designed, and supervised the study and wrote the paper.

ACKNOWLEDGMENTS

We thank all members of the Reik lab for helpful discussions, Felix Krueger for bioinformatics support, the sequencing facilities at Babraham Institute (BI) and Sanger Institute, and the flow cytometry facility at BI for support. We thank Jon Houseley, Andrea Schorn, and Rob Martienssen for helpful discussions, and Dónal O'Carroll for providing the AGO2 antibody and sharing the AGO2 IP protocol. F.v.M. was supported by the Swiss National Science Foundation. R.V.B. is funded by the Gates Cambridge Trust. W.R. is supported by the BBSRC (BB/K010867/1), Wellcome Trust (095645/Z/11/Z), EU BLUEPRINT, and EpiGeneSys. W.R. is a consultant and shareholder of CEGX.

Received: December 23, 2016
Revised: August 14, 2017
Accepted: October 12, 2017
Published: November 2, 2017

REFERENCES

Aravin, A.A., Sachidanandam, R., Bourc'his, D., Schaefer, C., Pezic, D., Toth, K.F., Bestor, T., and Hannon, G.J. (2008). A piRNA pathway primed by individual transposons is linked to de novo DNA methylation in mice. Mol. Cell *31*, 785–799.

Babiarz, J.E., Ruby, J.G., Wang, Y., Bartel, D.P., and Blelloch, R. (2008). Mouse ES cells express endogenous shRNAs, siRNAs, and other Microprocessor-independent, Dicer-dependent small RNAs. Genes Dev. *22*, 2773–2785.

Bodak, M., Cirera-Salinas, D., Yu, J., Ngondo, R.P., and Ciaudo, C. (2017). Dicer, a new regulator of pluripotency exit and LINE-1 elements in mouse embryonic stem cells. FEBS Open Bio *7*, 204–220.

Chen, C.Y., Morris, Q., and Mitchell, J.A. (2012). Enhancer identification in mouse embryonic stem cells using integrative modeling of chromatin and genomic features. BMC Genomics *13*, 152.

Creyghton, M.P., Cheng, A.W., Welstead, G.G., Kooistra, T., Carey, B.W., Steine, E.J., Hanna, J., Lodato, M.A., Frampton, G.M., Sharp, P.A., et al. (2010). Histone H3K27ac separates active from poised enhancers and predicts developmental state. Proc. Natl. Acad. Sci. USA *107*, 21931–21936.

Cruz, C., and Houseley, J. (2014). Endogenous RNA interference is driven by copy number. eLife *3*, e01581.

Doi, N., Zenno, S., Ueda, R., Ohki-Hamazaki, H., Ui-Tei, K., and Saigo, K. (2003). Short-interfering-RNA-mediated gene silencing in mammalian cells requires Dicer and eIF2C translation initiation factors. Curr. Biol. *13*, 41–46.

Ferguson-Smith, A.C. (2011). Genomic imprinting: the emergence of an epigenetic paradigm. Nat. Rev. Genet. *12*, 565–575.

Fire, A., Xu, S., Montgomery, M.K., Kostas, S.A., Driver, S.E., and Mello, C.C. (1998). Potent and specific genetic interference by double-stranded RNA in Caenorhabditis elegans. Nature *391*, 806–811.

Flemr, M., Malik, R., Franke, V., Nejepinska, J., Sedlacek, R., Vlahovicek, K., and Svoboda, P. (2013). A retrotransposon-driven dicer isoform directs endogenous small interfering RNA production in mouse oocytes. Cell *155*, 807–816.

Ghildiyal, M., and Zamore, P.D. (2009). Small silencing RNAs: an expanding universe. Nat. Rev. Genet. *10*, 94–108.

Goodier, J.L., and Kazazian, H.H., Jr. (2008). Retrotransposons revisited: the restraint and rehabilitation of parasites. Cell *135*, 23–35.

Haig, D. (2016). Transposable elements: Self-seekers of the germline, team-players of the soma. BioEssays *38*, 1158–1166.

Hajkova, P., Ancelin, K., Waldmann, T., Lacoste, N., Lange, U.C., Cesari, F., Lee, C., Almouzni, G., Schneider, R., and Surani, M.A. (2008). Chromatin dynamics during epigenetic reprogramming in the mouse germ line. Nature *452*, 877–881.

Han, B.W., Wang, W., Zamore, P.D., and Weng, Z. (2015). piPipes: a set of pipelines for piRNA and transposon analysis via small RNA-seq, RNA-seq, degradome- and CAGE-seq, ChIP-seq and genomic DNA sequencing. Bioinformatics *31*, 593–595.

Hutnick, L.K., Huang, X., Loo, T.-C., Ma, Z., and Fan, G. (2010). Repression of retrotransposal elements in mouse embryonic stem cells is primarily mediated by a DNA methylation-independent mechanism. J. Biol. Chem. *285*, 21082–21091.

Illingworth, R.S., and Bird, A.P. (2009). CpG islands–'a rough guide'. FEBS Lett. *583*, 1713–1720.

Iurlaro, M., von Meyenn, F., and Reik, W. (2017). DNA methylation homeostasis in human and mouse development. Curr. Opin. Genet. Dev. *43*, 101–109.

Karimi, M.M., Goyal, P., Maksakova, I.A., Bilenky, M., Leung, D., Tang, J.X., Shinkai, Y., Mager, D.L., Jones, S., Hirst, M., and Lorincz, M.C. (2011). DNA methylation and SETDB1/H3K9me3 regulate predominantly distinct sets of genes, retroelements, and chimeric transcripts in mESCs. Cell Stem Cell *8*, 676–687.

Kelley, D., and Rinn, J. (2012). Transposable elements reveal a stem cell-specific class of long noncoding RNAs. Genome Biol. *13*, R107.

Kobayashi, H., Sakurai, T., Miura, F., Imai, M., Mochiduki, K., Yanagisawa, E., Sakashita, A., Wakai, T., Suzuki, Y., Ito, T., et al. (2013). High-resolution DNA methylome analysis of primordial germ cells identifies gender-specific reprogramming in mice. Genome Res. *23*, 616–627.

Krueger, F., and Andrews, S.R. (2011). Bismark: a flexible aligner and methylation caller for Bisulfite-Seq applications. Bioinformatics *27*, 1571–1572.

Lander, E.S., Linton, L.M., Birren, B., Nusbaum, C., Zody, M.C., Baldwin, J., Devon, K., Dewar, K., Doyle, M., FitzHugh, W., et al.; International Human Genome Sequencing Consortium (2001). Initial sequencing and analysis of the human genome. Nature *409*, 860–921.

Langmead, B., and Salzberg, S.L. (2012). Fast gapped-read alignment with Bowtie 2. Nat. Methods *9*, 357–359.

Lei, H., Oh, S.P., Okano, M., Jüttermann, R., Goss, K.A., Jaenisch, R., and Li, E. (1996). De novo DNA cytosine methyltransferase activities in mouse embryonic stem cells. Development *122*, 3195–3205.

Lerat, E., Rizzon, C., and Biémont, C. (2003). Sequence divergence within transposable element families in the Drosophila melanogaster genome. Genome Res. *13*, 1889–1896.

Love, M.I., Huber, W., and Anders, S. (2014). Moderated estimation of fold change and dispersion for RNA-seq data with DESeq2. Genome Biol. *15*, 550.

Maksakova, I.A., Romanish, M.T., Gagnier, L., Dunn, C.A., van de Lagemaat, L.N., and Mager, D.L. (2006). Retroviral elements and their hosts: insertional mutagenesis in the mouse germ line. PLoS Genet. *2*, e2.

Murchison, E.P., Partridge, J.F., Tam, O.H., Cheloufi, S., and Hannon, G.J. (2005). Characterization of Dicer-deficient murine embryonic stem cells. Proc. Natl. Acad. Sci. USA *102*, 12135–12140.

Ran, F.A., Hsu, P.D., Wright, J., Agarwala, V., Scott, D.A., and Zhang, F. (2013). Genome engineering using the CRISPR-Cas9 system. Nat. Protoc. *8*, 2281–2308.

Reik, W., and Surani, M.A. (2015). Germline and pluripotent stem cells. Cold Spring Harb. Perspect. Biol. *7*, a019422.

Robert, V.J.P., Sijen, T., van Wolfswinkel, J., and Plasterk, R.H.A. (2005). Chromatin and RNAi factors protect the C. elegans germline against repetitive sequences. Genes Dev. *19*, 782–787.

Rowe, H.M., and Trono, D. (2011). Dynamic control of endogenous retroviruses during development. Virology *411*, 273–287.

Schorn, A.J., Gutbrod, M.J., LeBlanc, C., and Martienssen, R. (2017). LTR-retrotransposon control by tRNA-derived small RNAs. Cell *170*, 61–71.e11.

Seisenberger, S., Andrews, S., Krueger, F., Arand, J., Walter, J., Santos, F., Popp, C., Thienpont, B., Dean, W., and Reik, W. (2012). The dynamics of genome-wide DNA methylation reprogramming in mouse primordial germ cells. Mol. Cell *48*, 849–862.

Sharif, J., Endo, T.A., Nakayama, M., Karimi, M.M., Shimada, M., Katsuyama, K., Goyal, P., Brind'Amour, J., Sun, M.A., Sun, Z., et al. (2016). Activation of endogenous retroviruses in Dnmt1(-/-) ESCs involves disruption of SETDB1-mediated repression by NP95 binding to hemimethylated DNA. Cell Stem Cell *19*, 81–94.

Stadler, M.B., Murr, R., Burger, L., Ivanek, R., Lienert, F., Schöler, A., van Nimwegen, E., Wirbelauer, C., Oakeley, E.J., Gaidatzis, D., et al. (2011). DNA-binding factors shape the mouse methylome at distal regulatory regions. Nature *480*, 490–495.

Svoboda, P., Stein, P., Anger, M., Bernstein, E., Hannon, G.J., and Schultz, R.M. (2004). RNAi and expression of retrotransposons MuERV-L and IAP in preimplantation mouse embryos. Dev. Biol. *269*, 276–285.

Tam, O.H., Aravin, A.A., Stein, P., Girard, A., Murchison, E.P., Cheloufi, S., Hodges, E., Anger, M., Sachidanandam, R., Schultz, R.M., and Hannon, G.J. (2008). Pseudogene-derived small interfering RNAs regulate gene expression in mouse oocytes. Nature *453*, 534–538.

Tang, W.W.C., Kobayashi, T., Irie, N., Dietmann, S., and Surani, M.A. (2016). Specification and epigenetic programming of the human germ line. Nat. Rev. Genet. *17*, 585–600.

Trapnell, C., Pachter, L., and Salzberg, S.L. (2009). TopHat: discovering splice junctions with RNA-Seq. Bioinformatics *25*, 1105–1111.

van de Lagemaat, L.N., Medstrand, P., and Mager, D.L. (2006). Multiple effects govern endogenous retrovirus survival patterns in human gene introns. Genome Biol. *7*, R86.

von Meyenn, F., Iurlaro, M., Habibi, E., Liu, N.Q., Salehzadeh-Yazdi, A., Santos, F., Petrini, E., Milagre, I., Yu, M., Xie, Z., et al. (2016). Impairment of DNA methylation maintenance is the main cause of global demethylation in naive embryonic stem cells. Mol. Cell *62*, 848–861.

Walsh, C.P., Chaillet, J.R., and Bestor, T.H. (1998). Transcription of IAP endogenous retroviruses is constrained by cytosine methylation. Nat. Genet. *20*, 116–117.

Walter, M., Teissandier, A., Pérez-Palacios, R., and Bourc'his, D. (2016). An epigenetic switch ensures transposon repression upon dynamic loss of DNA methylation in embryonic stem cells. eLife *5*, e11418.

Watanabe, T., Takeda, A., Tsukiyama, T., Mise, K., Okuno, T., Sasaki, H., Minami, N., and Imai, H. (2006). Identification and characterization of two novel classes of small RNAs in the mouse germline: retrotransposon-derived siRNAs in oocytes and germline small RNAs in testes. Genes Dev. *20*, 1732–1743.

Yoshimizu, T., Sugiyama, N., De Felice, M., Yeom, Y.I., Ohbo, K., Masuko, K., Obinata, M., Abe, K., Schöler, H.R., and Matsui, Y. (1999). Germline-specific expression of the Oct-4/green fluorescent protein (GFP) transgene in mice. Dev. Growth Differ. *41*, 675–684.

STAR★METHODS

KEY RESOURCES TABLE

REAGENT or RESOURCE	SOURCE	IDENTIFIER
Antibodies		
anti-CD4 microbead	Miltenyl Biotec	Cat #: 130-045-101
Alexa Fluor 647, goat anti-mouse IgG antibody	Thermo Fisher Scientific	Cat# A-21236; RRID: AB_141725
Alexa Fluor 568 donkey anti - rabbit IgG antibody	Thermo Fisher Scientific	Cat# A10042; RRID: AB_2534017
Rabbit Anti-Nanog Polyclonal Antibody, Unconjugated	Abcam	Cat# ab80892; RRID: AB_2150114
AGO2 antibody	Dr. O'Carrolls lab	N/A
Histone H3K9me3 antibody	Active Motif	Cat #: 61013; RRID: AB_2687870
H3K27me3-mouse antibody	Active Motif	Cat #: 39155; RRID: AB_2561020
Histone H3K9me2 antibody	Abcam	Cat #: ab1220; RRID: AB_449854
Bacterial and Virus Strains		
E.coli: One Shot TOP10 chemically competent cells	Thermo Fisher Scientific	Cat #: K450001
Chemicals, Peptides, and Recombinant Proteins		
Tamoxifen	Sigma-Aldrich	Cat #: T5648-1G
mouse LIF	Stem Cell Institute, Cambridge	N/A
Na/Deoxycholate	Sigma-Aldrich	Cat #: D6750-10G
N-lauroylsarcosine	Sigma-Aldrich	Cat #: 61739-5G
Vanadyl ribonucleoside complex	New England Biolabs	Cat #: S1402S
Lipofectamine 2000	Thermo Fisher Scientific	Cat #: 11668027
Protein G-coupled Dynabeads	Thermo Fisher Scientific	Cat #: 10003D
HiFi Uracil+ ReadyMix	KAPABiosystems	Cat #: KK2801
T4 RNA Ligase 2, truncated	New England Biolabs	Cat #: M0242S
Tri-Reagent	Sigma-Aldrich	Cat #: T9424-200ML
Phenol/chloroform/isoamylalcohol (25:24:1)	Life Technologies	Cat #: 15593031
Triton X-100	Sigma-Aldrich	Cat #: RES9690T
Dimethylsulfoxide (DMSO)	Thermo Fisher Scientific	Cat #: TS-20684
Ampicillin	Sigma-Aldrich	Cat #: A9518-5G
Penicillin/Streptomycin	Thermo Fisher Scientific	Cat #: 15140122
L-glutamine	Thermo Fisher Scientific	Cat #: 25030081
Non-essential amino acids	Thermo Fisher Scientific	Cat #: 11140050
2-Mercaptoethanol (50mM)	Life technologies	Cat #: 31350-010
RNase A	Thermo Fisher Scientific	Cat #: EN0531
cOmplete Protease Inhibitor Cocktail	Sigma-Aldrich	Cat #: 00000001169 7498001
Proteinase K	Thermo Fisher Scientific	Cat #: EO0491
Paraformaldehyde 16% Solution	Agar Scientific	Cat #: AGR1026
Gelatine	Sigma-Aldrich	Cat #: G9391
DTT	Sigma-Aldrich	Cat #: D0632-1G
Fetal Bovine Serum (FBS)	Stem Cell Institute, Cambridge	N/A
DMEM (High Glucose) w/L-Glutamine andamp; Na Pyr	Life Technologies	Cat #: 41966-052
NEBuffer 2	New England Biolabs	Cat #: B7002S
Trypsin EDTA (1x) 100ml	Life technologies	Cat #: 25300-054
HyperLadder 1kb, 100bp	Bioline	Cat #: BIO-33053, BIO-33029
SYBR Safe	Invitrogen	Cat #: S33102
SYBR Gold	Life Technologies	Cat #: S11494

(*Continued on next page*)

Continued

REAGENT or RESOURCE	SOURCE	IDENTIFIER
PvuI	New England Biolabs	Cat #: R0150S
EcoRI HF	New England Biolabs	Cat #: R3101L
T4 Polynucleotide Kinase	New England Biolabs	Cat #: M0201L
T4 Ligase	New England Biolabs	Cat #: M0202T
Ampure XP beads	Beckman Coulter	Cat #: A63880
T5 Exonuclease	New England Biolabs	Cat #: M0363S
Exonuclease I	New England Biolabs	Cat #: M0293S
Klenow exo-	New England Biolabs	Cat #: M0212L
Glycoblue	Ambion	Cat #: AM9516
Optimem	GIBCO	Cat #: 31985062
DAPI	Thermo Fisher Scientific	Cat #: 62248
MyTaq Redmix	Bioline	Cat #: BIO-25043
Orange G dye	Sigma-Aldrich	Cat #: 861286-25G
Critical Commercial Assays		
TruSeq Small RNA Library Prep Kit -Set A (24 rxns) (Set A-c: indexes 1-36)	Illumina	Cat #:RS-200-0012, RS-200-0024, RS-200-0036
NEBNext DNA Library Prep Master Mix Set for Illumina	New England Biolabs	Cat #: E6040S
Imprint DNA Modification Kit	Sigma-Aldrich	Cat #: MOD50-1KT
TruSeq RNA library preparation kit v2	Illumina	Cat #: RS-122-2001
MicroPlex Library Preparation kit	Diagenode	Cat #: C05010012
SmallRNA qRTPCR miRNA kit: mmu_miR93	Taqman	Cat #: TM001090
SmallRNA qRTPCR miRNA kit: mmu_miR7081_mat	Taqman	Cat #: TM467052_mat
SmallRNA qRTPCR miRNA kit: snoRNA202	Taqman	Cat #: 001232
Dharmacon siGENOME SMARTpool, mouse Dicer	Dharmacon	Cat #: MU-040892-01-0005
Dharmacon siGENOME SMARTpool, mouse Dgcr8	Dharmacon	Cat #: MU-051365-00-0002
Dharmacon siGENOME SMARTpool, mouse Ago2	Dharmacon	Cat #: MU-058989-01-0005
Dharmacon siGENOME SMARTpool, mouse Dicer	Dharmacon	Cat #: D-001210-02-05
Miniprep kit	QIAGEN	Cat #: 27106
Gel extraction kit	GeneJET	Cat #: K0691
PCR Purification kit	GeneJET	Cat #: K0701
Qiaamp DNA micro kit	QIAGEN	Cat #: 56304
TURBO DNA-free kit	Life Technologies	Cat #: AM1907
Quant-iT PicoGreen dsDNA Assay kit	Life Technologies	Cat #: P11496
Platinum SYBR Green qPCR SuperMix-UDG w/ROX	Life Technologies	Cat #: 11744100
QuickExtract	Epicenter	Cat #: QE09050
Kapa Library Quantification kit	Kapa Biosystems	Cat #: KK4847
High Sensitivity DNA kit	Agilent	Cat #: 5067-4626
High Sensitivity total RNA kit	Agilent	Cat #: 5067-1513
Deposited Data		
Raw and analyzed data	This study	GEO: GSE89698
Mouse reference genome NCBI build 37, NCBIM37	Mouse Genome Sequencing Consortium	http://may2012.archive.ensembl.org/Mus_musculus/Info/Index
Mouse repeats	repeatmasker v4.0.3, library version 20130422	http://www.repeatmasker.org/
Mouse ESCs enhancer annotation track	Chen et al., 2012; Creyghton et al., 2010	N/A
CpG island promoters	Illingworth and Bird, 2009	N/A
Promoters: regions −1kb to the transcription start site	Ensemble, NCBIM37 version 67	N/A

(*Continued on next page*)

Continued		
REAGENT or RESOURCE	SOURCE	IDENTIFIER
Experimental Models: Cell Lines		
Dnmt1 cKO: Passage 12 Dnmt1$^{loxP/loxP}$ (C57BL/6) ESCs	Sharif et al., 2016	N/A
Dicer/Dnmt1 cDKO: Passage 21 Dicer $^{loxP/loxP}$/ Dnmt1 $^{loxP/loxP}$ ESCs	This study	See STAR Methods section CRISPR cKO and KO
Ago2/Dnmt1 cDKO: Passage 21 Ago2 $^{loxP/loxP}$ /Dnmt1 $^{loxP/loxP}$ ES cells	This study	See STAR Methods section CRISPR cKO and KO
Dicer KO: Passage 17 *Dicer* KO/*Dnmt1* $^{loxP/loxP}$ ES cells	This study	See STAR Methods section CRISPR cKO and KO
Ago2 KO: Passage 17 *Ago2* KO/*Dnmt1* $^{loxP/loxP}$ ES cells	This study	See STAR Methods section CRISPR cKO and KO
Experimental Models: Organisms/Strains		
Mouse: C57BL/6J female mice carrying the Oct4-GFP transgene in the developing gonad: B6.Cg-Tg(GOF18/EGFP)11Ymat/Rbrc	Yoshimizu et al., 1999	RRID: IMSR_RBRC00868
Oligonucleotides		
Primers for CRISPR clone generation, see Table S3	This paper	N/A
Primers for RTqPCR clone generation, see Table S2	This paper	N/A
Recombinant DNA		
Cas9 plasmid: pSpCas9(BB)-2A-GFP	Ran et al., 2013	Addgene Plasmid #48138
pSpCas9(BB)-2A-hCD4	This study	N/A
Software and Algorithms		
Bowtie2	Langmead and Salzberg, 2012	http://bowtie-bio.sourceforge.net/bowtie2/index.shtml
Bismark	Krueger and Andrews, 2011	https://www.bioinformatics.babraham.ac.uk/projects/bismark/, version 0.14.4
TopHat	Trapnell et al., 2009	http://ccb.jhu.edu/software/tophat/index.shtml
piPipes	Han et al., 2015	https://github.com/bowhan/piPipes/wiki
Trim Galore	N/A	http://www.bioinformatics.babraham.ac.uk/projects/trim_galore/, Version 0.4.1
SeqMonk software	N/A	http://www.bioinformatics.babraham.ac.uk/projects/seqmonk/
DESeq2	Love et al., 2014	https://bioconductor.org/packages/release/bioc/html/DESeq2.html, version 3.5
Transposon analysis	this study	STAR Methods section Transposon analysis
R	Data analysis	https://www.r-project.org/, version 3.2.5
Adobe Illustrator	Figures	http://www.adobe.com/de/products/illustrator.html, version CC 2015.3

CONTACT FOR REAGENT AND RESOURCE SHARING

Further information and requests for resources and reagents should be directed to and will be fulfilled by the Lead Contact, Rebecca Berrens (rebecca.berrens@gmail.com). The AGO2 antibody was obtained from EMBL, after establishing an MTA with the laboratory of Prof. Donal O'Carroll at University of Edinburgh.

EXPERIMENTAL MODEL AND SUBJECT DETAILS

Cell lines

Mouse embryonic stem cell (ESC) lines were used in this study. *Dnmt1*$^{loxP/loxP}$ ESCs (strain C57BL/6) were obtained from Haruhiko Koseki, RIKEN Center for Integrative Medical Sciences, Yokohama City, Japan (Sharif et al., 2016). *Dicer/Dnmt1* cDKO, *Ago2/Dnmt1* cDKO, *Dicer* KO and *Ago2* KO ESC lines were generated using *Dnmt1*$^{loxP/loxP}$ ESCs using the CRISPR/Cas9 targeting and screening primers mentioned in Table S3.

Mice

All *in vivo* PGC samples were collected from timed matings of C57Bl/6J male with C57BL/6J female mice carrying the Oct4-GFP transgene expressed in the developing gonad (Yoshimizu et al., 1999). Primordial germ cells from male and female embryos at E13.5 and E14.5 were collected. All procedures were covered by a project license (to WR) under the Animal (Scientific Procedures) Act 1986, and are locally regulated by the Babraham Institute Animal Welfare, Experimentation, and Ethics Committee.

METHOD DETAILS

DNA/RNA Extraction

Genomic DNA was prepared using QIAmp DNA Micro Kit (QIAGEN). RNA was extracted using TriReagent (Sigma) and Phase Lock tubes (5Prime) following manufacturers' instructions and subjected to DNase treatment using the DNA-free kit (Ambion DNA-free DNA Cat #1311027) according to the manufacturers' instructions.

Small RNA Quantitative Real-Time PCR

For small RNA qPCR Taqman miRNA kits were used according to the manufacturer's' instructions for mmu_miR93 (Taqman, Cat. No. TM001090), mmu_miR7081_mat (Taqman, Cat. No. TM467052_mat) and snoRNA202 (Taqman, Cat. No. 001232) was used as a positive control. Quantitative real-time PCR primers are listed in Table S2.

AGO2 IP

ESCs were cultured on 15 cm dishes and harvested in 1 x PBS. Pellets were frozen at −80°C until further processing. ESC were resuspended in 300 μl Lysis buffer (50 mM Tris, pH8, 150 mM NaCl, 5 mM $MgCl_2$, 15% Glycerol, 1 mM DTT, 0.5% Sodium deoxycholate, 0.5% Triton X-100, Protease inhibitor cocktail (Roche), 50μg/ml yeast tRNA, 2mM Vanadyl ribonucleoside complex) and cells were pelleted at 10,000 rpm, 10 min, 4°C. The supernatant was used as whole ESC extract. 25 μL beads (protein G Sepharose) were washed 3 times with 1 mL of Wash Buffer (10 mM Tris pH 8, 150 mM NaCl, 1 mM $MgCl_2$, 0,01% NP-40). 50 μl of purified AGO2 antibody (O'Carroll lab) was added, filled up to 1mL with Wash Buffer and incubated O/N at 4°C in a rotating wheel. On the next day, the beads were washed 3 times with Wash Buffer and the negative control (beads with extract but without serum) was prepared. The ESC extract was pre-spun to remove precipitated proteins and 200μL extract was added to the beads and filled up to 600μL with Lysis buffer. The mix was incubated for 2-4h at 4°C in a rotating wheel and subsequently washed 5 times with wash buffer and the IP was eluted with 300μL Proteinase K buffer (10 mM Tris pH 7,5, 0,5% SDS, 5 mM EDTA, 1 μL Proteinase K/reaction) after 30 min for 50°C incubation on the thermomixer, at 850 rpm. RNA was isolated by phenol extraction and eluted in 8 μl H_2O.

RNAi knockdown of Ago2, Dicer1, Dgcr8 in Dnmt1fl/fl ES cells

RNA interference experiments were performed according to manufacturers' instructions with modifications. Transfections of Dharmacon siGENOME SMARTpool siRNA against mouse *Dicer* (Dharmacon, Cat. No. MU-040892-01-0005), *Dgcr8* (Dharmacon, Cat. No. MU-051365-00-0002) or *Ago2* (Dharmacon, Cat. No. MU-058989-01-0005) and siGENOME non-targeting siRNA#2 (Dharmacon, Cat. No. D-001210-02-05) were performed with Lipofectamine 2000 according to the manufacturers' instructions. The transfection was done in two rounds. The cells were plated at a density of 1 × 10^5 ES cells per well of gelatinized 12-well plate. One day later the first transfection was done the following for each well of a 12 well plate: 50uM siRNA were added to 100 μl DMEM. 6 μl of Lipofectamin2000 were mixed with 100 μl DMEM. The mix was incubated for 5 min at room temperature. Afterward the two solutions were mixed and incubated at room temperature for 15 min. 200 μl of the siRNA and Lipofectamin2000 mix were added to each well of a 12 well plate. On the third day the medium was changed. On the fourth day the second transfection was done the following: 125uM siRNA were added to 250 μl DMEM. 7.5 μl of Lipofectamin2000 were added to 250 μl DMEM and incubated at room temperature for 5 min. The solutions were then mixed and again incubated for 15 min at room temperature. The cells were washed with PBS, trypsinized, inactivated and resuspended in ESC medium and plated on a gelatinized 6-well plate I a total volume of 1.8 mL each well. 500μl of siRNA and Lipofectamin2000 were added to each well. The ESCs were incubated at 37C for 6 hours and then the medium was changed.

Cells were harvested 48 h after the 2nd transfection and RNA was extracted and analyzed.

Quantitative Real-Time PCR

100 ng −1 μg of DNase treated RNA was reverse transcribed (Thermo RevertAid #K1622) using random hexamer primers. Endogenous controls (*Atp5b*, *Hspcb*, *U1*) were used to normalize expression. Primers are listed in Table S2.

CRISPR cKO and KO

guideRNAs (gRNAs) were constructed following https://chopchop.rc.fas.harvard.edu/ and http://crispr.mit.edu/ and cloned following the protocol by Ran et al. (2013) into pSpCas9(BB)-2A-GFP (Addgene plasmid ID: 48138) or pSpCas9(BB)-2A-hCD4, constructed by replacing the GFP in the pSpCas9(BB)-2A-GFP with human CD4. Cells were cultured on feeder plates and transfected with 1 μg gRNA and 100 ng donor DNA, where appropriate, using Lipofectamine 2000 transfection reagent. Cells were sorted for GFP in single cell colonies into 96 well plates using flow cytometry or CD4 expression plating on 10cm dishes as single cell colonies.

Colonies were screened by PCR using MyTaq (Bioline, BIO-25044) and Sanger sequencing. See Figure S3 for knock out strategy and Table S3 for gRNAs, screening primers and donor DNA sequence.

Fluorescence-activated cell sorting (FACS)

Cells were trypsinized and resuspended in PBS + 1% FBS and analyzed on a LSR Fortessa Cell Analyzer (BD). Cells were gated for singlets and living cells were identified using the level of DAPI incorporation and the level of GFP signal was recorded for each cell.

CD4 pull down

Cells were trypsinized and resuspended in 70 μl 1 x PBS and stained with human CD4 Microbead antibody (Miltenyl Biotec, Cat. No. 130-045-101) according to manufacturers' instructions. The CD4 positive cells were enriched using MACS columns. Negative cells were collected from flow through. The cells were eluted in 500 μl 1 x PBS.

In vivo PGC collection

All embryonic samples for library preparation were collected from timed mattings of C57BL/6J female mice PGCs that express the Oct4-GFP transgene in the developing gonad (Yoshimizu et al., 1999). E13.5 and E14.5 PGCs, male and female samples were collected separately and after collagenase digestion PGC samples were sorted for GFP positive cells using a FACSAria (BD) cell sorter with > 98% purity.

Cell lines and culture conditions

Mouse ESCs were cultured with or without feeders on gelatinized plates (0.1% gelatin) in serum-containing media (DMEM 4,500 mg/l glucose, 4 mM L-glutamine, 15% fetal bovine serum, 1 U/ml penicillin, 1 μg/ml streptomycin, 0.1 mM nonessential amino acids, 50 μM β-mercaptoethanol) supplemented with mouse LIF at 37°C and 5% CO_2. Conditional deletion was induced by Cre mediated recombination, as described before (Sharif et al., 2016). Cre expression was induced in response to tamoxifen (4OHT, 800 nM).

WGBS-seq libraries

For preparation of WGBS-seq libraries, genomic DNA was sonicated using a Covaris Sonicator, followed by end-repair, A-tailing and methylated adaptor (Illumina) ligation using NEBNext reagents (E6040S, NEB). Afterward the libraries were bisulfite treated using Imprint DNA modification kit (MOD50-1KT, Sigma), followed by library amplification with indexed primers using KAPA HiFi Uracil Hot-Start DNA Polymerase (KAPA HiFi Uracil+, KK2801/2). Subsequently, the amplified libraries were purified and assessed for quality and quantity using High-Sensitivity DNA chips on an Agilent Bioanalyzer. High-throughput sequencing of all libraries was carried out with a 75 bp or 50 bp paired-end (PE) sequencing on Illumina HiSeq 2500 instruments using TruSeq reagents (Illumina, San Diego, CA, USA), according to manufacturers' instructions.

ChIP-seq libraries

ESCs were grown on 15 cm dishes coated with 0.1% gelatine until they were 80% confluent. Subsequently cells were cross-linked with 1% methanol-free formaldehyde in fresh medium for 10 minutes. To quench the cross-linking, 0.2 M final concentration of glycine was added. ESCs were washed twice with ice cold 1 x PBS (137 mM NaCl, 2.7 mM KCl, 10 mM Na2HPO4, 2 mM KH2PO4 dissolved in 800 mL distilled H2O, pH was adjusted to 7.4 with HCl) and harvested using a cell scraper. Cells were then pelleted by centrifugation at 8,000 x g at 4 ∘C for 3 min. Pellets were resuspended in LB1 buffer (50 mM HEPES' KOH, pH 7.5; 140 mM NaCl; 1 mM EDTA; 10% glycerol; 0.5% NP-40; 0.25% Triton X-100, protease inhibitors) for 10 minutes at 4°C, pelleted and resuspended in LB2 buffer (10 mM Tris/HCl, pH 8.0; 200 mM NaCl; 1 mM EDTA; 0.5 mM EGTA, protease inhibitors) for 10 minutes at 4 ∘C. Cells were pelleted and resuspended in LB3 buffer (10 mM Tris-HCl, pH 8; 100 mM NaCl; 1 mM EDTA; 0.5 mM EGTA; 0.1% Na/Deoxycholate; 0.5% N-Lauroylsarcosine, protease inhibitors). Next the cells were sonicated using Misonix Sonicator 3000. Triton X-100 was added to a final concentration of 1% and the lysate was centrifuged at 20,000 x g for 10 min to pellet the debris. The bead-antibody complexes were prepared before adding the sonicated DNA. Protein G-coupled Dynabeads (Thermo Fisher Scientific, Cat. No. 10003D) and the primary antibodies in PBS with 5 mg/ml BSA were incubated ON. Subsequently, the bead-antibody complexes were added to the sonicated chromatin and both were incubated at 4 ∘C ON. On the following day, beads were washed extensively with RIPA buffer (50 mM HEPES pH 7.6, 1 mM EDTA, 0.7% Na deoxycholate, 1% NP-40, 0.5M LiCl), once with 1x TE bu er (1 M Tris-HCl (pH approximately 8.0), 0.1 M EDTA) and eluted in 200 μL of buffer containing 1% SDS and 0.1 M NaHCO3. They were then incubated at 65°C ON for reverse cross-linking. RNase A treatment at 37°C was carried out for 1 h, then Proteinase K treatment at 55°C for 2 h. The DNA was then extracted with phenol/chloroform, followed by ethanol precipitation. ChIP-seq library preparation was performed using MicroPlex Library Preparation kit (Diagenode) following manufacturer's instructions. Libraries were quantified using the High Sensitivity DNA Bioanalyzer kit and Kapa library quantification. High-throughput sequencing of all libraries was carried out with a 100 bp PE sequencing on Illumina HiSeq 2500 instruments.

Small RNA-seq libraries

Small RNA-seq libraries were produced according to the Illumina protocol (RS-200-0012), with the following changes: 10 ng or 1 μg RNA (RIN of 8-10) were used as input material. The instructions were followed until the cDNA purification. In order to purify the cDNA, the samples were run on 10% Novex PAGE gel. The entire area between the 145 and 160 bp markers was excised, gel purified by

addition of 0.3 M NaCl and the DNA was eluted from the gel by rotation over night at 4°C. The DNA was precipitated in EtOH overnight and the library was quantified using the HighSensitivity Bioanalyzer kit. The small RNA-seq libraries were additionally quantified by Kapa Library Quantification. The libraries were pooled according to their molecular weight. High-throughput sequencing of all libraries was carried out with a 50 bp SE on Miseq or SE and PE on Illumina HiSeq 2500 instruments.

Total RNA-seq libraries

Stranded Total RNaseq libraries were prepared according to manufacturers' protocols using the Illumina stranded Total RNaseq library preparation after Ribo-zero depletion. High-throughput sequencing of all libraries was carried out with a 100 bp PE on Illumina HiSeq 2500 instruments.

QUANTIFICATION AND STATISTICAL ANALYSIS

WGBS-seq mapping and analysis

Raw sequence reads from WBGS libraries were trimmed to remove poor quality reads and adaptor contamination, using Trim Galore (v0.4.1, http://www.bioinformatics.babraham.ac.uk/projects/trim_galore/) with default parameters. The remaining sequences were mapped using Bismark (v0.14.4) (Krueger and Andrews, 2011) with default parameters to the mouse reference genome Ensembl v67 NCBIM37 in paired-end mode. Reads were then deduplicated and CpG methylation calls were extracted from the deduplicated mapping output using the Bismark methylation extractor (v0.14.4) in paired end mode. CpG methylation calls were analyzed using R and SeqMonk software (http://www.bioinformatics.babraham.ac.uk/projects/seqmonk/). The custom R scripts can be found in Data S1. Global CpG methylation levels of pooled replicates were calculated in windows of 50 CpGs with a coverage of at least 3, illustrated using bean plots. Methylation over a given genomic feature was calculated by averaging the individual methylation levels of CpGs covered by at least 3 reads and only features with at least 50 CpGs were used. Promoters were defined as the region −1 kb to the transcription start site as annotated in Ensembl NCBIM37 v67. For analysis of specific genome features these were defined as follows: Gene bodies (probes overlapping genes), CGI promoters (promoters containing a CGI) (Illingworth and Bird, 2009), non-CGI promoters (all other promoters).

RNA-seq mapping and analysis

RNA-seq sequences were trimmed using Trim Galore using default settings. Trimmed sequencing reads were aligned to mouse genome assembly NCBIM37 using TopHat (Trapnell et al., 2009) and reads with MAPQ scores < 20 were discarded. Mapped RNA-seq data were quantitated using the RNA-seq quantitation pipeline in SeqMonk software to generate log2 RPM (reads per million reads of library) expression values. Genes were considered to be differentially expressed if they were significantly different ($p < 0.05$ after Benjamini and Hochberg multiple testing correction) when analyzed with both DESeq2 and Intensity difference (SeqMonk) statistical tests.

Global pervasive transcription, was calculated as following: Genes with significant antisense expression were identified by initially counting both sense and antisense reads over all genes in the genome. A global expected antisense level was defined by the total proportion of antisense reads across all genes. Individual genes were considered to show significant antisense expression if they had a binomial p value < 0.05 following multiple testing correction (FDR) using the global antisense proportion as the expected success rate, the total reads for that gene as the trials and the total antisense reads for that gene as successes. Additionally, the raw antisense transcription counts for all samples was calculated and significant differential antisense expression was calculated using DESeq2 with an FDR < 0.05. The overlap of the two quantifications was used to define pervasive transcription, and the difference in antisense transcription between WT and KO samples at each time point was plotted using R.

ChIP-seq mapping and analysis

ChIP-seq sequencing data was trimmed to remove poor quality reads, adaptor and barcodes sequences using Trim Galore. Trimmed data were mapped using Bowtie2 (Langmead and Salzberg, 2012) against the mouse reference genome Ensembl v67 NCBIM37 and reads with a MAPQ value < 20 were discarded. Mapped ChIP-seq data were quantitated creating 1kb tiles of the whole genome and calculating the log2 observed/expected value comparing the observed read count with the expected count had all reads been uniformly distributed over the genome.

Small RNA-seq mapping and analysis

For small RNA-seq data analysis trimmed sequencing reads were filtered to 20-24nt length and mapped to the mouse NCBIM37 genome assembly using Bowtie2. Raw overlap counts for each small RNA molecule were quantitated using SeqMonk. Graphing and statistics was performed using Excel or R. For consensus sequence mapping the piPipes small RNA pipeline was used (https://github.com/bowhan/piPipes) (Han et al., 2015). IAPEZ consensus sequences were used from repeatmasker libraries (repeatmasker v4.0.3, library version 20130422). Additionally, the small RNA-seq data processing was performed using the freely available piRNA pipeline piPipes. For repeat mapping, trimmed data were mapped using Bowtie2 against repeats as defined in the analysis by using the mouse repeatmasker annotation. The plots shown were generated as described below: The distribution of small RNAs was computed by mapping all small RNA-seq reads to the individual genomic features. The length distribution was calculated taking all uniquely mapped small RNAs into account, excluding small RNA-seq mapping to ribosomal RNAs (rRNAs). For all subsequent

analysis, small RNA reads were pre-filtered as follows: reads mapping to rRNAs and miRNAs were excluded, then reads aligning to the repeat masked mm9 genome (all annotated repeats were masked/replaced by Ns) were removed, too. The remaining small RNAs reads were mapped to the mouse repeatmasker annotation. The 5′ end nucleotide composition was computed from the uniquely mapped small RNAs. Similarly, analysis of the position of 5′ to 5′ overlap was performed on the mapped small RNAs reads and the length distribution and strand orientation of small RNAs shown was generated using uniquely mapped small RNA reads.

Transposon analysis

Repeat locations for a pre-defined set of repeat classes of interest were extracted from the pre-masked repeatmasker 4.0.3-20130422 library in the mm9 genome. Repeat instances within 2 kb of an annotated gene in the Ensembl v67 NCBIM37 gene set were removed to avoid mixing signals from genic expression with specific expression of repetitive sequences. RNA-seq data were processed and mapped as described above (RNA-seq mapping and analysis). We set a standard outlier filtering approach with a cutoff of counts > 3. Overlaps were quantitated between the mapped RNA-seq reads and the repeat instances. This allowed an unbiased identification of TEs depending on *Dnmt1 KO* as well as *Dicer KO*, which we followed throughout this manuscript. Summed counts for all instances of each class of repeat were calculated and these were corrected for both the total length of all TEs and the size of the individual libraries to generate log2 RPM expression values. The matrix of expression values and samples were plotted using the R pheatmap library allowing the repeat classes to cluster using default parameters. WGBS-seq libraries were processed and mapped as described above (WGBS-seq mapping and analysis). Methylation levels at the repeat instances were quantitated by summing up all methylation calls and non-methylation calls for all instances of each class of repeat and calculating the percentage of methylated Cs over all Cs. Only TEs with at least 1000 observations in all samples were used for the analysis and calculation of percentage methylation. For major satellite methylation analysis Bismark (Krueger and Andrews, 2011) was used to map all reads against the mouse major satellite consensus sequence (GSAT from repeatmasker) and the methylation calls from these results were analyzed directly. The custom R scripts can be found in Data S1.

Statistics

Statistical values including the exact number of replicates (n), the definition of standard deviation and statistical significance are reported in the Figure Legends.

WGBS-seq

For statistical analysis WGBS-seq of Figures 1B and S1 of WT versus *Dnmt1* KO data we used the Wilcoxon rank sum test with Bonferroni correction testing with a p value threshold of < 0.05. The code of the analysis of the retained methylation over TEs can be found in Data S1.

Total RNA-seq

To call differentially expressed mRNAs, we applied the SeqMonk intensity difference filter with Benjamini and Hochberg correction for multiple testing with a p value threshold of < 0.05 and overlapped them with the genes called differentially expressed by DESeq2 with a p value threshold of < 0.05 and multiple testing correction.

For TE analysis we only considered significantly differentially expressed TEs p < 0.05 of Dnmt1 KO over WT samples into account. The code of the analysis can be found in Data S1.

small RNA-seq

To call differentially expressed miRNAs we overlapped the differentially expressed miRNAs using DESeq2 with multiple testing correction and SeqMonk intensity difference filter with Benjamini and Hochberg correction with a p value of < 0.05.

To call differential amount of mapped small RNAs to TEs we used Students t test to compare day 8 to day 0 enrichment of small RNAs with a p value of < 0.05.

ChIP-seq

As we only have data from one measurement we could not call significant differences of histone modification enrichment but show TEs which have at least 2 times higher enrichment in Dnmt1 KO versus WT samples. The code of the analysis can be found in Data S1.

Quantitative Real-Time PCR

Each quantitative real-time PCR was done with 3 technical replicates. Differences between conditions that are statistically significant are denoted by *p value < 0.05, **p value < 0.005 using the standard distributed two tailed t test.

siRNA knock-down

Every siRNA knock-down was done in 3 technical replicates. Differences between conditions that are statistically significant are denoted by *p value < 0.05, ** p value < 0.005 using the standard distributed two tailed t test.

DATA AND SOFTWARE AVAILABILITY

The accession number for the next-generation-sequencing data reported in this study is GEO: GSE89698. The software of this study can be found in Data S1.

Cell Stem Cell, Volume *21*

Supplemental Information

An endosiRNA-Based Repression Mechanism Counteracts Transposon Activation during Global DNA Demethylation in Embryonic Stem Cells

Rebecca V. Berrens, Simon Andrews, Dominik Spensberger, Fátima Santos, Wendy Dean, Poppy Gould, Jafar Sharif, Nelly Olova, Tamir Chandra, Haruhiko Koseki, Ferdinand von Meyenn, and Wolf Reik

Supplemental Information

Supplemental Figure Legends

Figure S1. Global DNA demethylation and transcriptional change upon acute Dnmt1 deletion, Related to Figure 1

(A) WGBS-seq reads overlapping the whole Chromosome 2 between WT (day 1-day 11) and *Dnmt1* cKO ESCs induced for 1-11 days. Percentage of methylated cytosines were counted for each consecutive 50 CpG window genome-wide.

(B) Enrichment of CpG methylation over transcription starts sites (TSS) and gene body in WT and *Dnmt1* cKO ESCs induced 1 day (dark red), 3 days (light red), 6 days (light pink), 9 days (light blue), 11 days (dark blue). Measurement of 2 biological replicates. Percentage of methylated cytosines were counted for each consecutive 50 CpG window genome-wide.

(C) Bean plots showing distribution of methylation levels for genome features between WT (gray) and conditional *Dnmt1* cKO ESC induced for 1 day (dark red), 3 days (light red), 6 days (light pink), 9 days (light blue), 11 days (dark blue). Low methylated regions (LMRs) (Stadler et al., 2011), enhancers defined by H3K4m1 (Chen et al., 2012) and H3K27ac (Creyghton et al., 2010). Measurement of 2 biological replicates. For significance analysis Wilcoxon rank sum test with Bonferroni correction testing with a p-value threshold of < 0.05 was used.

(D) Chromosome view of RNA-seq reads over mRNA with Lx5 or MIRb TE sitting in the 2kb surrounding region of a coding gene. RNA-seq libraries are strand specific. Each read is depicted.

(E) Violin plots showing distribution of methylation levels for different TE classes between WT (gray) and conditional *Dnmt1* cKO ESC induced for 1 day (dark red), 3 days (light red), 6 days (light pink), 9 days (light blue), 11 days (dark blue). Measurement of 2 biological replicates. For significance analysis Wilcoxon rank sum test with Bonferroni correction testing with a p-value threshold of < 0.05 was used.

(F) Graphs showing methylation retention of TE classes in comparison to the rest of the genome. Left: scatter plot of WGBS sequencing reads in gradient of gray with specific TE class as red dot, Right: Line graph of TE class in time course (red) in comparison to probes starting with the same methylation level as the respective TE class (blue) and in comparison, to the rest of the genome (gray). Measurement of 2 biological replicates.

(G) Scatter plot of all reads overlapping genes in the genome with the significantly *Dnmt1* responsive genes highlighted in black. Significance was called by combining both Intensity difference (SeqMonk) as well as DESeq2 significance called genes with a p-value threshold of < 0.05 and multiple testing correction.

(H) Venn Diagram of the number and overlap of mRNAs upregulated upon *Dnmt1* cKO.

(I) Bar graph of 6 genes in WT which were most highly upregulated and downregulted upon *Dnmt1* cKO induced 0 days (black), 1 day (dark red), 3 days (light red), 6 days (light pink), 9 days (light blue), 11 days (dark blue). Dots show the expression level in the 2 RNA-seq libraries for each time point.

(J) Bar plots of expression of key pluripotency genes between WT (gray) and conditional *Dnmt1* cKO ESC not induced (black), induced for 1 day (dark red), 3 days (light red), 6 days (light pink), 9 days (light blue). Measurements of 2 biological replicate shown next to each other.

(K) Bar plot showing percentage of genic insertions of *Dnmt1* and *Dicer* responsive TEs in sense (red) and antisense (blue) direction to the respective genes.

Figure S2. Genome wide small RNA response upon Dnmt1 conditional KO, Related to Figure 2

(A) Bar plots of small RNA size distribution as well as classification of different small RNA classes in *Dnmt1* cKO and WT ESCs mapped to the whole genome; miRNAs (gray), rRNA (green), small nuclear RNAs (snRNAs) (violet), miscellaneous other RNAs (misc RNAs) (red), small nucleolar RNAs (snoRNA) (orange) and tRNA (light blue) of WT (right) and after conditional *Dnmt1* cKO (left).

(B) Expression of endogenously transcribed miRNAs in WT (gray) and in conditional *Dnmt1* cKO induced for 1 day (dark red), 3 days (light red), 6 days (light pink), 9 days (light blue), 11 days (dark blue). Error bars represent mean +/-SD of 3 technical replicates.

(C) Genic location of miRNA 200c with reads mapped in Dnmt1 cKO and WT ESCs, each line representing one read.

(D) Scatter plot of all small RNAs in the genome, highlighting miRNAs of the *Dlk* cluster (black) and *Xlr3* cluster (green) at day 9 after *Dnmt1* cKO (y-axis) versus WT (x-axis). Significance was called by combining both Intensity difference (SeqMonk) as well as DESeq2 significance called genes with a p-value threshold of < 0.05 and multiple testing correction.

(E) Bar graph of 2 representative small RNAs of the *Xlr3* and *Dlk* locus in WT and upon *Dnmt1* cKO induced 1 day (dark red), 3 days (light red), 6 days (light pink), 9 days (light blue), 11 days (dark blue). Error bars represent mean +/-SD of 3 technical replicates. Statistics: two-sided Students t-test, * p-value <0.05, ** p-value <0.005, *** p-value <0.0005.

(F) Confirmation of small RNA-seq data by small RNA quantitative real-time PCR analysis. Bar plot showing small RNA quantitative real-time PCR analysis of mmu-miR-543 and mmu-miR-367 in WT (gray) and conditional *Dnmt1* cKO induced for 9 days (dark red). Error bars represent mean +/-SD of 3 technical replicates. Statistics: two-sided Students t-test, * p-value <0.05, ** p-value <0.005, *** p-value <0.0005.

(G) Chromosome view of WGBS-seq, total RNA-seq and small RNA-seq depicted as wiggle plots overlapping imprinted control regions (ICR), mRNA and small RNAs in WT and at day 9 after *Dnmt1* deletion.

(H) Pie chart distribution showing mapping of small RNA-seq from AGO2 IP 9 days after conditional *Dnmt1* to different small RNA classes. miRNAs (black), repeats (dark green), 3'UTRs (yellow), introns (dark blue), piRNAs (light blue), 5'UTRs (light green), others (gray).

(I) Bar plot showing nucleotide position 30 nt upstream and downstream of 5' end of AGO2 IP small RNA-seq libraries mapping to repeats after conditional *Dnmt1* cKO induced 9 days.

(J) Bar plot showing small RNA duplex 5' to 5' overlap of AGO2 IP small RNA-seq mapping to repeats after conditional *Dnmt1* cKO induced 9 days.

(K) Small RNA-seq of 20-24 nt small RNAs mapped to TEs *in vivo* PGCs of E13.5 as well as E14.5 male (blue) and female (red) PGCs. Each library was done as 1 replicate.

(L) Pie chart distribution of small RNAs mapping to different genomic loci of *in vivo* E14.5 male PGC small RNA-seq libraries after conditional *Dnmt1* cKO induced 9 days. miRNAs (black), repeats (dark green), 3'UTRs (yellow), introns (dark blue), 5'UTRs (light green), rRNA_tRNA (gray), unannotated (white).

(M) Size distribution for *in vivo* E14.5 male PGCs of sense (blue) and antisense (red) small RNAs mapping to repeatmasker consensus sequences using piPipes small RNA pipeline.

(N) Bar plot showing siRNA duplex 5' to 5' overlap for *in vivo* E14.5 male PGC small RNA-seq libraries mapping to repeats.

(O) Bar plot showing nucleotide position 30 nt upstream and downstream of 5' end of *in vivo* E14.5 male PGC small RNA-seq library mapping to repeats.

Figure S3. Characterisation of the involvement DICER and AGO2 in TE silencing, Related to Figure 3

(A) Left: Schematic showing *Dicer* cKO generation using CRISPR by introducing loxP sites into Intron 14_15 and Intron 20_21. Agarose gel of PCR to screen for genomic recombination of 2 *Dicer/Dnmt1* conditional double cKO clones after addition of 4OHT for 3 days. Recombination of Intron 15-16 was tested with primer set 1, recombination of intron 20-21 was tested with primer set 2 and recombination of both introns was tested with primer set 3, LD = 1000 bp DNA ladder. Middle: Quantitative real-time PCR analysis of *Dicer* mRNA upon CRE recombination induced by tamoxifen (4OHT) in clone 1 (light green) and clone 2 (dark green) of *Dicer* conditional KO ESCs. Error bars represent mean +/-SD of 3 technical replicates. Statistics: two-sided Students t-test, * p-value <0.05, ** p-value <0.005, *** p-value <0.0005. Right: Quantitative real-time PCR analysis of *mmu-miR-93* expression in ESCs upon *Dicer* KO in clone 1 (light green) and clone 2 (dark green) controlled by snoRNA expression. Error bars represent mean +/-SD of 3 technical replicates. Statistics: two-sided Students t-test, * p-value <0.05, ** p-value <0.005.

(B) Bar graph of percentage of genic antisense transcription over the time course of *Dicer/Dnmt1* cDKO*, Dicer* KO *and Dnmt1* cKO in KO over WT samples. Measurement of 2 biological replicates for *Dicer/Dnmt1* cDKO *and Dnmt1* cKO and WT samples and 1 replicate for *Dicer* KO ESCs.

(C) Bar plots of small RNA size distribution as well as classification of different small RNA classes in *Dicer/Dnmt1* cDKO, *Dnmt1* cKO with KO induced for 4 days and *Dnmt1*$^{fl/fl}$ mESCs and WT mapped to the whole genome; miRNAs (light blue), rRNA (gray), small nuclear RNAs (snRNAs) (dark blue), miscellaneous other RNAs (misc RNAs) (orange), small nucleolar RNAs (snoRNA) (yellow) and tRNA (light green).

(D) Small RNA-seq of Dicer/Dnmt1 cDKO and Dnmt1 cKO ESCs normalised to WT ESCs mapped to IAPEz and L1MdGf TE classes. *p<0.05, **p<0.005, two-tailed student t-test. Measurement of 2 biological replicates.

(E) Schematic showing *Ago2* cKO generation using CRISPR by introducing loxP sites into Intron 8_9 and Intron 11_12 of *Ago2* mRNA. Agarose gel of PCR to screen for genomic recombination of four *Ago2/Dnmt1* cDKO clones after addition of 4OHT for 3 days in comparison to one WT clone. Recombination of Intron 8-12 was tested with primer set 1. LD = 100 bp DNA ladder.

(F) Quantitative real-time PCR analysis analysis of *Ago2* in ESCs following *Ago2/Dnmt1* cDKO by treatment with 4OHT or control (EtOH) for 3 days. Error bars represent mean +/-SD of 3 biological replicates in 3 technical replicates. Values were normalized to *Hspcb* and controlled to EtOH samples. Statistics: two-sided Students t-test, * p-value <0.05, ** p-value <0.005.

(G) Immunofluorescence of AGO2 protein (purple) in *Ago2/Dnmt1* cDKO and *Dnmt1* cKO ESCs upon KO induction with 4OHT. Deletion was induced for 3 or 8 days as depicted. Nuclear DAPI counter staining (white). scale bar = 20µm.

(H) Upper panel: Schematic knock out strategy for *Dicer* in mouse ESCs constructing gRNAs against Exon 23 and 24 of Dicer mRNA. gRNA Protospacer Adjacent Motif (PAM) sequences (dark blue). (Bernstein et al., 2003). Lower left: Quantitative real-time PCR analysis of mRNA expression of *Dicer* in WT (black) and *Dicer* cKO (dark blue). Error bars represent mean +/- standard deviations of 3 technical replicates. Statistics: two-sided Students t-test, * p-value <0.05, ** p-value <0.005, *** p-value <0.0005. Lower right: Expression level of *mmu-miR-93* in wildtype (black) and *Dicer* cKO (dark blue). Error bars represent mean +/-standard deviations of 3 technical replicates. Statistics: two-sided Students t-test, * p-value <0.05, ** p-value <0.005, *** p-value <0.0005,

(I) Upper panel: Schematic of knock out strategy for *Ago2* in mouse ESCs constructing gRNAs against Intron 13-14 and 115 of Dicer mRNA. gRNA PAM sequences (light green). Lower panel: Quantitative real-time PCR analysis of Ago2 expression in 2 clones of Ago2 KO

ESCs (dark purple) in comparison to Dnmt1$^{fl/fl}$ ESCs (black). Error bars represent mean +/- standard deviations of 3 technical replicates. Statistics: two-sided Students t-test, * p-value <0.05, ** p-value <0.005, *** p-value <0.0005,

(J) Immunofluorescence of AGO2 protein (purple) and NANOG (green) in *Ago2* KO*/Dnmt1* cKO and mouse embryonic fibroblasts. Nuclear DAPI counter staining (white). scale bar = 20μm.

(K) Bar plots of expression of 5 pluripotency genes between WT (gray) and conditional *Dnmt1* cKO ESC induced for 11 days (dark blue), Dicer KO (light blue) treated with EtOH for 1 day and 11 days, Dicer KO/Dnmt1 DKO (faint blue) treated with 4OHT for 1 and 11 days.

(L) Scatter plot of RNA-seq data of *Dicer* KO (y-axis) versus WT (x-axis) ESCs. Differentially expressed genes were called by intensity difference of SeqMonk (black), all other genes are depicted in gray.

(M) Chromosome view of read count quantitation across the 4 genes *Lin28*, *Dnmt3l*, *Fbln2* and *Oct4*. High bars indicated high expression, low bars indicate low expression. Every bar overlaps at least 1 read.

(N) Quantitative real-time PCR analysis data of LINE and major satellites in *Dicer* KO/*Dnmt1* cKO following conditional *Dnmt1* cKO, by treatment with 4OHT. Error bars represent SD of 3 technical replicates. Values were normalized to *Atp5b*, *Hspcb* and Major satellites to U1. Error bars represent mean +/-standard deviations of 3 technical replicates. Statistics: two-sided Students t-test, * p-value <0.05, ** p-value <0.005, *** p-value <0.0005,

(O) Heatmap of unbiased hierarchical clustering of all TE classes responsive *Dicer* KO/*Dnmt1* cKO versus *Dnmt1* cKO. Heatmap is showing relative expression (z-score) of TEs upon *Dnmt1* cKO and were generated using the pheatmap R library.

Figure S4. Distribution of repressive histone marks – H3K9me3, H3K9me2 and H3K27me3 in ESCs upon Dnmt1 cKO, Related to Figure 4

(A) Pie chart of enrichment of H3K27me3, H3K9me3 and H3K9me2 in repeats (dark violet), genic regions (light violet), promoters (dark green), CGIs (middle green), intergenic regions (light green) in wildtype ESCs.

(B) Probe enrichment of H3K9me3 (green), H3K9me2 (yellow) and H3K27me3 (blue) over gene body and TSS in wildtype ESCs.

(C) Aligned probe plot of H3K27me3 enrichment surrounding 5kb of TSS in wildtype ESCs.

(D) Scatter plot of repressive histone marks overlapping genes in wildtype (y-axis) versus *Dnmt1* cKO (x-axis) ESCs.

(E) Read count plots of ChIP enrichment of H3K9me3 (green), H3K27me3 (blue) and H3K9me2 (yellow) over a 500kbp region in Chromosome 12. Intensity of the enrichment on the y-axis. Plots were generated using SeqMonk read count quantitation.

(F) Read count plots of H3K9me3 enrichment over IAPEZ in *Dnmt1* cKO at day 4 (red), day 8 (blue) and in WT (gray). Plots were generated using SeqMonk read count quantitation.

(G) Bar graph of enrichment of H3K27me3, H3K9me3 and H3K9me2 in repeats (dark violet), genic regions (light violet), promoters (dark green), CGIs (middle green), intergenic regions (light green) in WT ESCs, *Dnmt1* cKO, *Dicer* KO and *Dicer/Dnmt1* cDKO

(H) Read count plots of ChIP-seq enrichment of H3K9me3, H3K27me3 and H3K9me2 at three genomic loci in *Dicer/Dnmt1* cDKO at day 11 (light blue), Dicer KO (middle blue), *Dnmt1* cKO at day 11 (dark blue) and WT (gray). Enrichment intensity shown on y-axis. Plots were generated using SeqMonk read count quantitation.

(I) Summary of TE classes across WGBS-seq, RNA-seq, small RNA-seq and ChIP-seq libraries. Scale from red (loose) to green (gain).

Supplemental Tables

Table S1: List of differentially expressed genes upon Dnmt1 KO and Dicer KO, Related to Figure 1 and S1 and Figure 3 and S3.

Differentially expressed genes were called using the overlap between the SeqMonk Intensity difference as well as DESeq2.

Table S2: Quantitative real-time PCR analysis primers, Related to Figure 3 and S3

Primers below have been used for expression analyses (Quantitative real-time PCR analysis primers).

Table S3: CRISPR primers, Related to Figure 3, S3

CRISPR primers were used to construct *Dicer* KO/*Dnmt1* cKO and *Dicer/Dnmt1* cDKO, *Ago2* KO/*Dnmt1* cKO and *Ago2/Dnmt1* cDKO mouse ES cells. gRNA (guide RNA).

Supplemental Data

Data S1: Raw code to analyse TEs, Related to Figure 1-4.

Figure S1

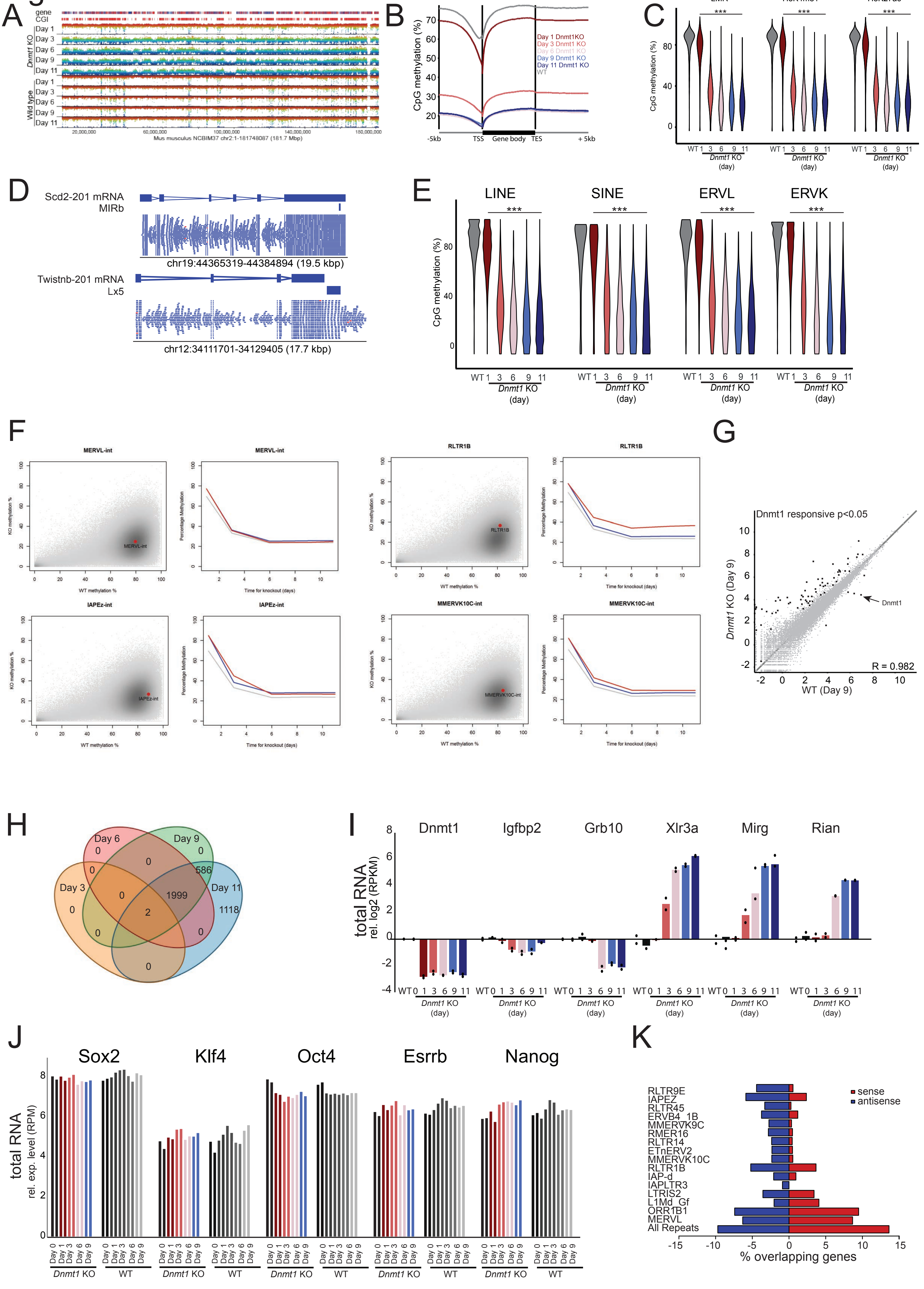

A
gene
CGI
Dnmt1 KO
Wild type
Day 1
Day 3
Day 6
Day 9
Day 11
20,000,000
60,000,000
100,000,000
140,000,000
180,000,000
Mus musculus NCBIM37 chr2:1-181748087 (181.7 Mbp)
B
CpG methylation (%)
Day 1 Dnmt1KO
Day 3 Dnmt1 KO
Day 6 Dnmt1 KO
Day 9 Dnmt1 KO
Day 11 Dnmt1 KO
WT
-5kb
TSS
Gene body
TES
+ 5kb
C
LMR
H3K4me1
H3K27ac
***
CpG methylation (%)
WT 1 3 6 9 11
Dnmt1 KO
(day)
D
Scd2-201 mRNA
MIRb
chr19:44365319-44384894 (19.5 kbp)
Twistnb-201 mRNA
Lx5
chr12:34111701-34129405 (17.7 kbp)
E
LINE
SINE
ERVL
ERVK
CpG methylation (%)
F
MERVL-int
RLTR1B
IAPEz-int
MMERVK10C-int
KO methylation %
WT methylation %
Percentage Methylation
Time for knockout (days)
G
Dnmt1 responsive p<0.05
Dnmt1
Dnmt1 KO (Day 9)
WT (Day 9)
R = 0.982
H
Day 3
Day 6
Day 9
Day 11
1999
586
1118
I
Dnmt1
Igfbp2
Grb10
Xlr3a
Mirg
Rian
total RNA
rel. log2 (RPKM)
WT 0 1 3 6 9 11
Dnmt1 KO
(day)
J
Sox2
Klf4
Oct4
Esrrb
Nanog
total RNA
rel. exp. level (RPM)
Day 0
Day 1
Day 3
Day 6
Day 9
Dnmt1 KO
WT
K
sense
antisense
RLTR9E
IAPEZ
RLTR45
ERVB4_1B
MMERVK9C
RMER16
RLTR14
ETnERV2
MMERVK10C
RLTR1B
IAP-d
IAPLTR3
LTRIS2
L1Md_Gf
ORR1B1
MERVL
All Repeats
% overlapping genes

Figure S2

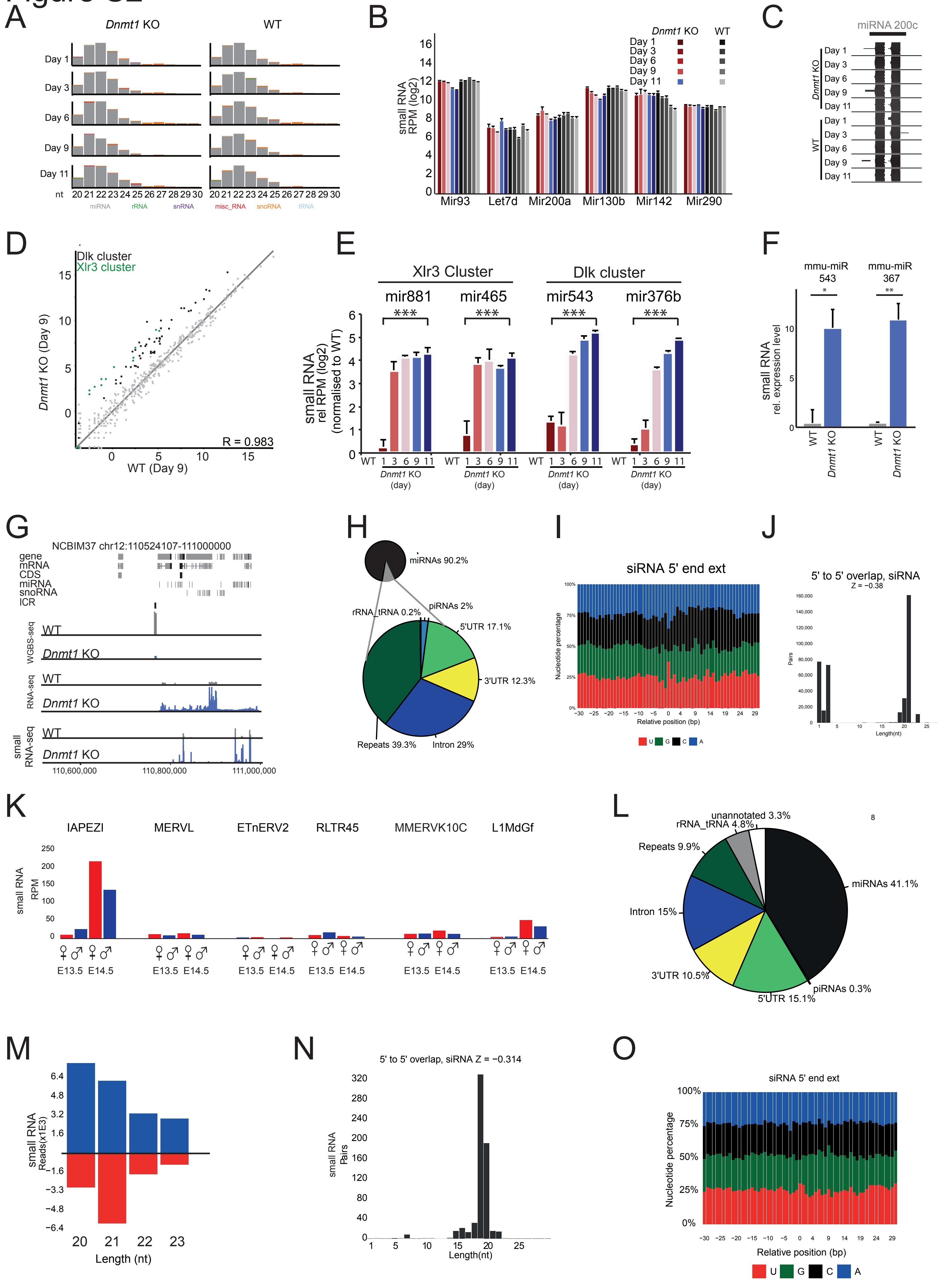
A
Dnmt1 KO
WT
Day 1
Day 3
Day 6
Day 9
Day 11
nt
miRNA rRNA snRNA misc_RNA snoRNA tRNA
B
small RNA RPM (log2)
Mir93 Let7d Mir200a Mir130b Mir142 Mir290
C
miRNA 200c
D
Dlk cluster
Xlr3 cluster
Dnmt1 KO (Day 9)
WT (Day 9)
R = 0.983
E
Xlr3 Cluster
Dlk cluster
mir881 mir465 mir543 mir376b
small RNA rel RPM (log2) (normalised to WT)
Dnmt1 KO (day)
F
mmu-miR 543
mmu-miR 367
small RNA rel. expression level
G
NCBIM37 chr12:110524107-111000000
gene
mRNA
CDS
miRNA
snoRNA
ICR
WGBS-seq
RNA-seq
small RNA-seq
110,600,000 110,800,000 111,000,000
H
miRNAs 90.2%
rRNA_tRNA 0.2%
piRNAs 2%
5'UTR 17.1%
3'UTR 12.3%
Repeats 39.3%
Intron 29%
I
siRNA 5' end ext
Nucleotide percentage
Relative position (bp)
U G C A
J
5' to 5' overlap, siRNA
Z = −0.38
Pairs
Length(nt)
K
IAPEZI MERVL ETnERV2 RLTR45 MMERVK10C L1MdGf
small RNA RPM
E13.5 E14.5
L
unannotated 3.3%
rRNA_tRNA 4.8%
Repeats 9.9%
miRNAs 41.1%
Intron 15%
3'UTR 10.5%
piRNAs 0.3%
5'UTR 15.1%
M
small RNA Reads(x1E3)
Length (nt)
N
5' to 5' overlap, siRNA Z = −0.314
small RNA Pairs
Length(nt)
O
siRNA 5' end ext
Nucleotide percentage
Relative position (bp)
U G C A

Figure S3

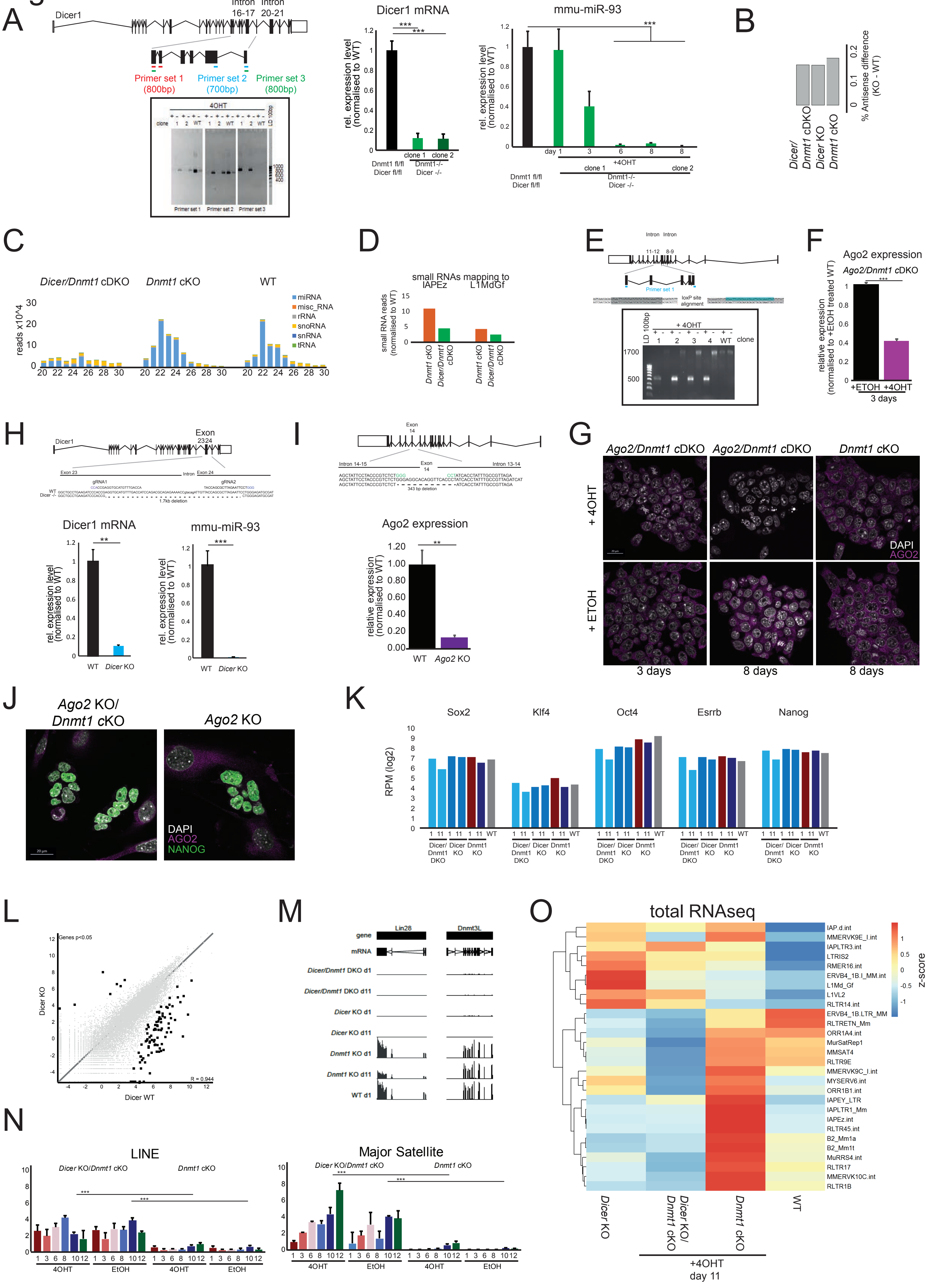

A
Dicer1
Intron 16-17
Intron 20-21
Primer set 1 (800bp)
Primer set 2 (700bp)
Primer set 3 (800bp)
4OHT
Dicer1 mRNA
rel. expression level (normalised to WT)
Dnmt1 fl/fl Dicer fl/fl
clone 1 clone 2 Dnmt1-/- Dicer -/-
mmu-miR-93
B
% Antisense difference (KO - WT)
Dicer/Dnmt1 cDKO
Dicer KO
Dnmt1 cKO
C
Dicer/Dnmt1 cDKO
Dnmt1 cKO
WT
reads x10^4
miRNA
misc_RNA
rRNA
snoRNA
snRNA
tRNA
D
small RNAs mapping to IAPEz L1MdGf
small RNA reads (normalised to WT)
E
loxP site alignment
F
Ago2 expression
Ago2/Dnmt1 cDKO
relative expression (normalised to +EtOH treated WT)
+ETOH +4OHT
3 days
H
Dicer1 mRNA
mmu-miR-93
WT Dicer KO
I
Ago2 expression
relative expression (normalised to WT)
WT Ago2 KO
G
Ago2/Dnmt1 cDKO
Ago2/Dnmt1 cDKO
Dnmt1 cKO
+ 4OHT
+ ETOH
DAPI
AGO2
3 days
8 days
8 days
J
Ago2 KO/ Dnmt1 cKO
Ago2 KO
DAPI
AGO2
NANOG
K
Sox2
Klf4
Oct4
Esrrb
Nanog
RPM (log2)
Dicer/Dnmt1 DKO
Dicer KO
Dnmt1 KO
L
Genes p<0.05
Dicer KO
Dicer WT
R = 0.944
M
Lin28
Dnmt3L
gene
mRNA
Dicer/Dnmt1 DKO d1
Dicer/Dnmt1 DKO d11
Dicer KO d1
Dicer KO d11
Dnmt1 KO d1
Dnmt1 KO d11
WT d1
N
LINE
Dicer KO/Dnmt1 cKO
Dnmt1 cKO
4OHT
EtOH
Major Satellite
O
total RNAseq
IAP.d.int
MMERVK9E_I.int
IAPLTR3.int
LTRIS2
RMER16.int
ERVB4_1B.I_MM.int
L1Md_Gf
L1VL2
RLTR14.int
ERVB4_1B.LTR_MM
RLTRETN_Mm
ORR1A4.int
MurSatRep1
MMSAT4
RLTR9E
MMERVK9C_I.int
MYSERV6.int
ORR1B1.int
IAPEY_LTR
IAPLTR1_Mm
IAPEz.int
RLTR45.int
B2_Mm1a
B2_Mm1t
MuRRS4.int
RLTR17
MMERVK10C.int
RLTR1B
z-score
Dicer KO
Dicer KO/ Dnmt1 cKO
Dnmt1 cKO
WT
+4OHT day 11

Figure S4

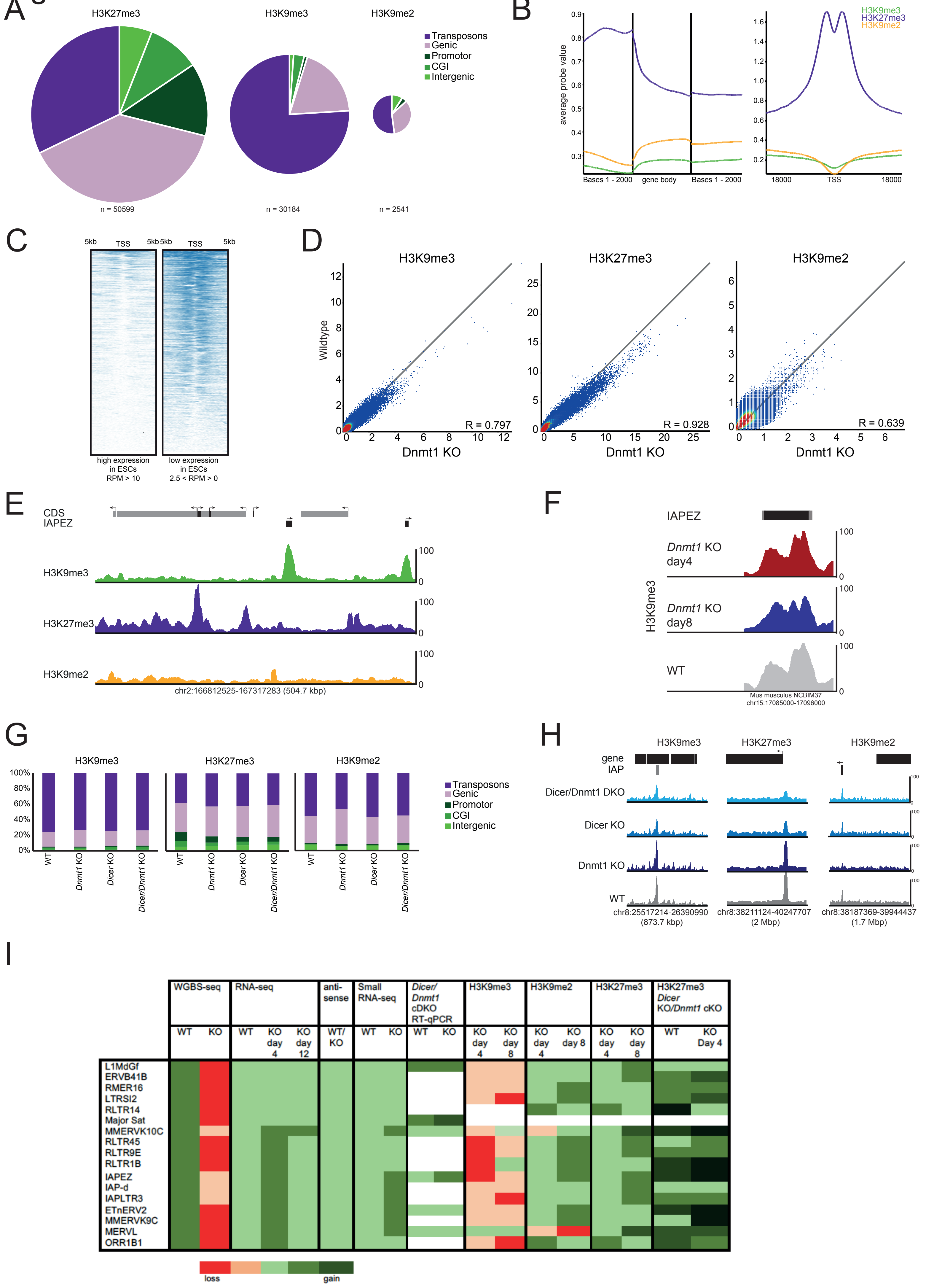
A
H3K27me3
H3K9me3
H3K9me2
Transposons
Genic
Promotor
CGI
Intergenic
n = 50599
n = 30184
n = 2541
B
average probe value
Bases 1 - 2000
gene body
Bases 1 - 2000
18000
TSS
18000
H3K9me3
H3K27me3
H3K9me2
C
5kb
TSS
5kb
high expression in ESCs RPM > 10
low expression in ESCs 2.5 < RPM > 0
D
H3K9me3
H3K27me3
H3K9me2
Wildtype
Dnmt1 KO
R = 0.797
R = 0.928
R = 0.639
E
CDS
IAPEZ
H3K9me3
H3K27me3
H3K9me2
chr2:166812525-167317283 (504.7 kbp)
F
IAPEZ
Dnmt1 KO day4
Dnmt1 KO day8
WT
H3K9me3
Mus musculus NCBIM37
chr15:17085000-17096000
G
H3K9me3
H3K27me3
H3K9me2
100%
80%
60%
40%
20%
0%
WT
Dnmt1 KO
Dicer KO
Dicer/Dnmt1 KO
Transposons
Genic
Promotor
CGI
Intergenic
H
H3K9me3
H3K27me3
H3K9me2
gene
IAP
Dicer/Dnmt1 DKO
Dicer KO
Dnmt1 KO
WT
chr8:25517214-26390990 (873.7 kbp)
chr8:38211124-40247707 (2 Mbp)
chr8:38187369-39944437 (1.7 Mbp)
I
WGBS-seq
RNA-seq
anti-sense
Small RNA-seq
Dicer/Dnmt1 cDKO RT-qPCR
H3K9me3
H3K9me2
H3K27me3
H3K27me3 Dicer KO/Dnmt1 cKO
WT
KO
KO day 4
KO day 12
WT/KO
KO day 8
KO Day 4
L1MdGf
ERVB41B
RMER16
LTRSI2
RLTR14
Major Sat
MMERVK10C
RLTR45
RLTR9E
RLTR1B
IAPEZ
IAP-d
IAPLTR3
ETnERV2
MMERVK9C
MERVL
ORR1B1
loss
gain

Table S2: RT-qPCR primers, Related to Figure 3 and S3

Gene	Primer	Sequence	origin
Hspcb	msRT_Hspcb_FW	GCTGGCTGAGGACAAGGAGA	
	msRT_Hspcb_RV	CGTCGGTTAGTGGAATCTTCATG	
Atp5b	msRT_Atp5b_FW	GGCCAAGATGTCCTGCTGTT	
	msRT_Atp5b_RV	GCTGGTAGCCTACAGCAGAAGG	
Dicer	Dicer_RT_17_18_FW	GCATTCCTAGCACCAAGTATTCA	This study
	Dicer_RT_17_18_RV	GGAAGGAAATTTACTGAGTGGGG	This study
	Dicer_RT_FW	GAACGAAATGCAAGGAATGGA	
	Dicer_RT_RV	GGGACTTCGATATCCTCTTCTTTCTC	
Ago2	Eif2c2_FW	GCCGTCCTTCCCACTACCAC	
	Eif2c2_RV	GGTATTGACACAGAGCGTGTGC	
Dgcr8	Dgcr8_FW	CCTAAAGACAGTGAAGAACTGGAGTA	
	Dgcr8_RV	CATGGAGGATCTGATATGGAGAC	
IAP	IAP_Nature_qPCR_FW	AAGCAGCAATCACCCACTTTGG	(ref)
	IAP_Nature_qPCR_RV	CAATCATTAGATGTGGCTGCCAAG	(ref)
MERVL	MuERV-L gag_Jafar_FW	TTCTTCTAGACCTGTAACCAGACTCA	(Sharif et al., 2016)
	MuERV-L gag_Jafar_RV	TCCTTAGTAGTGTAGCGAATTTCCTC	(Sharif et al., 2016)
Etn	MusD_Nature_qPCR_FW	GTGGTATCTCAGGAGGAGTGCC	
	MusD_Nature_qPCR_RV	GGGCAGCTCCTCTATCTGAGTG	
U1	U1_AP_FW	CTTACCTGGCAGGGGAGATA	
	U1_AP_FW	CAGTCCCCCACTACCACAAA	
Maj. Sat.	MajSat_BL_FW	GACGACTTGAAAAATGACGAAATC	
	MajSat_BL_RV	CATATTCCAGGTCCTTCAGTGTGC	
MMERVK10C	MmERVK10C_FW	ATGTGAGCTAGCTGTTAAAGAAGGAC	
	MmERVK10C_RV	CTCTCTGTTTCTGACATACTTTCCTGT	
LINEI	LINE ORF2_JS_FW	GACATAGACTAACAAACTGGCTACACAAAC	(Sharif et al., 2016)
	LINE ORF2_JS_RV	GGTAGTGTCTATCTTTTTCTCTGAGATGAG	(Sharif et al., 2016)

Table S3: CRISPR primers, Related to Figure 3 and S3

Gene	Primer	Sequence (5'-3')	
U6	U6-Fwd	GAGGGCCTATTTCCCATGATTCC	PCR screen
Dicer KO / *Dnmt1* cKO	Dicer1_X23_gRNA_FW	CACCGAGTAATCAAAAGGACCAGCC	gRNA
	Dicer1_X23_gRNA_RV	AAACGGCTGGTCCTTTTGATTACTC	gRNA
	Dicer1_X24_gRNA_FW	CACCGTTACCAGCGCTTAGAATTCC	gRNA
	Dicer1_X24_gRNA_RV	AAACGGAATTCTAAGCGCTGGTAAC	gRNA
	Dicer_23_24_screen_FW	AGCAGTGCATTGCTGACAAGAG	PCR screen
	Dicer_23_24_screen_RV	CTTGTGGTAGTCATACTTCACAGCC	PCR screen
Dicer/Dnmt1 cDKO	Dicer_14_15_gRNA_FW	CACCGCACTCAGCATCGAGTCTCG	gRNA
	Dicer_14_15_gRNA_RV	AAACCGAGACTCGATGCTGAGTGCC	gRNA
	Dicer_20_21_gRNA_FW	CACCGAGCAATGATCCGGTCTCAGG	gRNA
	Dicer_20_21_gRNA_RV	AAACCCTGAGACCGGATCATTGCTC	gRNA
	Dicer_14_15_RV1	TGAAACCAGACTTCTTCAGCTCG	PCR screen
	Dicer_14_15_FW1	CCTTTCCCTCTTGCACATTTACCT	PCR screen
	Dicer_2021_FW1	GGTGTCAGATCACTTCCCGT	PCR screen
	Dicer_2021_RV1	TGACCAGAATAAGAAGGAGCGGA	PCR screen
	Dicer_20_21_donor_loxP	gacaaggaccactgtactgtttatccctgaagtagcagactagaccattgagatcttgtcaagttagagagcagcaagaattctATAACTTCGTATAGCATACATTATACGAAGTTATgagaccggatcattgctcctgtagcagtgatgctggaataggggtgagaatggatatagttcttctcaaaactaa	Donor DNA
	Dicer_14_15_donor_loxP	ggcaagaaaagacatttatttctggttgtggggttaaacaaagcagcagcagcagctcagaaggcactcagcatcgagtctATAACTTCGTATAATGTATGCTATACGAAGTTATcgatcgaagccagagctgcacactgcccaattttacctatgctgcttattacagttttatggaatatcaaaagtatttaaaatag	Donor DNA
Ago2 KO/ *Dnmt1* cKO	Ago2_13_14_gRNA_FW	CACCGCTGGTCTAATCATGATCTAA	gRNA
	Ago2_13_14_gRNA_RV	AAACTTAGATCATGATTAGACCAGC	gRNA
	Ago2_14_15_gRNA_FW	CACCGAAGCTATTCCTACCCGTCTC	gRNA
	Ago2_14_15_gRNA_RV	AAACGAGACGGGTAGGAATAGCTTC	gRNA
	Ago2_13_14_FW	AGGCTACCTTGATGGACATGG	PCR screen
	Ago2_14_15_RV	GATGGGTTTGGTGGTACATGC	PCR screen
Ago2/Dnmt1 cDKO	Ago2_8_9_gRNA_FW	CACCGGTTACCTACAAGTTGTGTG	gRNA
	Ago2_8_9_gRNA_RV	AAACCACACAACTTGTAGGTAACC	gRNA
	Ago2_11_12_gRNA_FW	CACCGGTTGGTCAGACGGGTCACCG	gRNA
	Ago2_11_12_gRNA_RV	AAACAGGGTGACTGCCATTTATGAC	gRNA
	Ago2_8_9_FW	CCTGCTCTTCTGGAGGCATTT	PCR screen
	Ago2_8_9_RV	CCTGCTCTTCTGGAGGCATTT	PCR screen
	Ago2_11_12_FW	GTCCAGGGTGTGTGGGACAT	PCR screen
	Ago2_11_12_RV	GCAACTTCCTCAGCTAATCCTCCA	PCR screen
	Ago2_8_9_donor_loxP	ctcactgtgcacaggtcaagcccagcagagtgccaccaaagctgtagatggtcctctttcatgccagggttacctacaagAtAtCgtATAACTTCGTATAGCATACATTATACGAAGTTATgtgtggttgactttggagtggtcccaccactagtcagggttggttctggcttgcgtactcagcctctgaaatctccct	Donor DNA
	Ago2_11_12_donor_loxP	tgttggtcagacgggtcaccggggttccaataccagcggttggcagcctttctctaacagagagcactcacccaggATAACTTCGTATAGCATACATTATACGAAGTTATgAAtTCgactgccatttatgagatgtgacaaggccagattaggtgtgagagaaaacagctcctgagactgtagaaacttcactgtctat	Donor DNA
